# Metabolic Plasticity Is an Essential Requirement of Acquired Tyrosine Kinase Inhibitor Resistance in Chronic Myeloid Leukemia

**DOI:** 10.3390/cancers12113443

**Published:** 2020-11-19

**Authors:** Miriam G. Contreras Mostazo, Nina Kurrle, Marta Casado, Dominik Fuhrmann, Islam Alshamleh, Björn Häupl, Paloma Martín-Sanz, Bernhard Brüne, Hubert Serve, Harald Schwalbe, Frank Schnütgen, Silvia Marin, Marta Cascante

**Affiliations:** 1 Department of Biochemistry and Molecular Biomedicine, Faculty of Biology, Universitat de Barcelona, 08028 Barcelona, Spain; contrerasmostazo@ub.edu; 2 Institute of Biomedicine of University of Barcelona, 08028 Barcelona, Spain; 3 Department of Medicine, Hematology/Oncology, University Hospital Frankfurt, Goethe-University, 60590 Frankfurt am Main, Germany; kurrle@med.uni-frankfurt.de (N.K.); b.haeupl@dkfz-heidelberg.de (B.H.); serve@em.uni-frankfurt.de (H.S.); schnuetgen@em.uni-frankfurt.de (F.S.); 4 German Cancer Consortium (DKTK), Partner Site Frankfurt/Mainz, and German Cancer Research Center (DKFZ), 69120 Heidelberg, Germany; alshamleh@nmr.uni-frankfurt.de (I.A.); Schwalbe@em.uni-frankfurt.de (H.S.); 5 Frankfurt Cancer Institute (FCI), Goethe University, 60590 Frankfurt am Main, Germany; B.Bruene@biochem.uni-frankfurt.de; 6 Biomedicine Institute of Valencia, IBV-CSIC, 46010 Valencia, Spain; mcasado@ibv.csic.es; 7 CIBER of Hepatic and Digestive Diseases (CIBEREHD), Institute of Health Carlos III (ISCIII), 28029 Madrid, Spain; pmartins@iib.uam.es; 8 Institute of Biochemistry I, Faculty of Medicine, Goethe-University Frankfurt, 60590 Frankfurt am Main, Germany; fuhrmann@med.uni-frankfurt.de; 9 Center for Biomolecular Magnetic Resonance, Institute of Organic Chemistry and Chemical Biology, Goethe-University, 60438 Frankfurt am Main, Germany; 10 “Alberto Sols” Biomedical Research Institute, CSIC-UAM, 28029 Madrid, Spain; 11 Project Group Translational Medicine and Pharmacology TMP, Fraunhofer Institute for Molecular Biology and Applied Ecology, 60596 Frankfurt am Main, Germany; 12 Metabolomics Node at Spanish National Bioinformatics Institute (INB-ISCIII-ES- ELIXIR), Institute of Health Carlos III (ISCIII), 28029 Madrid, Spain

**Keywords:** tyrosine kinase inhibitors, chronic myeloid leukemia, metabolic reprogramming, proteomics

## Abstract

**Simple Summary:**

Tyrosine kinase inhibitors (TKIs), such as imatinib, have become the standard initial treatment of choice for chronic myeloid leukemia (CML) patients. However, one obstacle to face is that a significant proportion of patients presents poor response to TKIs, or acquires resistance resulting in disease relapses. Mutations in BCR-ABL1 protein are a well described mechanism of resistance but other not well established mechanisms outside *BCR-ABL1* mutations are emerging as important in the acquisition of resistance. Abnormal metabolism of CML cells that acquire resistance to imatinib has been pointed out as a putative downstream key event, but deep studies aimed to unveil metabolic adaptations associated with acquired resistance are still lacking. Here, we perform an exhaustive study on metabolic reprogramming associated with acquired imatinib resistance and we identify metabolic vulnerabilities of CML imatinib resistant cells that could pave the way for new therapies targeting TKI failure.

**Abstract:**

Tyrosine kinase inhibitors (TKIs) are currently the standard chemotherapeutic agents for the treatment of chronic myeloid leukemia (CML). However, due to TKI resistance acquisition in CML patients, identification of new vulnerabilities is urgently required for a sustained response to therapy. In this study, we have investigated metabolic reprogramming induced by TKIs independent of BCR-ABL1 alterations. Proteomics and metabolomics profiling of imatinib-resistant CML cells (ImaR) was performed. KU812 ImaR cells enhanced pentose phosphate pathway, glycogen synthesis, serine-glycine-one-carbon metabolism, proline synthesis and mitochondrial respiration compared with their respective syngeneic parental counterparts. Moreover, the fact that only 36% of the main carbon sources were utilized for mitochondrial respiration pointed to glycerol-phosphate shuttle as mainly contributors to mitochondrial respiration. In conclusion, CML cells that acquire TKIs resistance present a severe metabolic reprogramming associated with an increase in metabolic plasticity needed to overcome TKI-induced cell death. Moreover, this study unveils that KU812 Parental and ImaR cells viability can be targeted with metabolic inhibitors paving the way to propose novel and promising therapeutic opportunities to overcome TKI resistance in CML.

## 1. Introduction

Chronic myeloid leukemia (CML) is a malignant clonal disorder of hematopoietic stem cells that results in increases in myeloid and erythroid cells and in platelets in peripheral blood [1]. The cause of this disease is in the vast majority of the cases a translocation between chromosomes 9 and 22 (Philadelphia chromosome) [2] resulting in the expression of a *BCR-ABL1* fusion gene encoding for a constitutively activated tyrosine kinase as the driving oncogene. Nowadays, CML is considered to be a controllable disease since the development of the BCR-ABL1 specific tyrosine kinase inhibitor (TKI) imatinib, which is considered the “gold standard” in CML therapy [3]. However, despite the impressive success obtained with standard dose of imatinib as first therapeutic strategy for CML patients in chronic phase, approximately 25% of patients ultimately develop resistance to imatinib [4,5]. Although second generation TKIs (i.e., dasatinib and nilotinib) have been developed to overcome imatinib resistance, TKI resistance is still a clinical problem [6]. The molecular mechanisms of imatinib resistance development are heterogeneous, involving BCR-ABL1 secondary mutations [7,8] or *BCR-ABL1* gene amplification [9], to the overexpression of multidrug resistance genes (e.g., P-glycoprotein) [10]. 

Metabolic reprogramming has been extensively described for different types of cancer [11,12], and emerging evidences suggest that it is strongly dependent on the tissue of origin and the tumor microenvironment [13]. Recent studies also demonstrate that rapid metabolic rewiring in cancer cells is also responsible for the occurrence of a relapse after chemotherapy, and can also mediate resistance to targeted cancer drugs [14]. 

Several studies have shown that imatinib exposure leads to alterations in glucose uptake, and in de novo nucleic acid and/or fatty acid synthesis in BCR-ABL1-positive cell lines [15,16,17]. Furthermore, it has also been shown in BCR-ABL1-positive cell lines that metabolic changes in the tricarboxylic acid (TCA) cycle are dose-dependent. Thus, low doses of imatinib lead to a decrease in lactate production an induction of this cycle, whereas high doses down-regulate it and induce apoptosis [15,18]. In addition, it has been reported that imatinib can induce cardiotoxicity due to mitochondrial alterations [19] and, in recent studies, suggested that the activity of complex I is inhibited upon imatinib treatment in C2C12 myoblast and human rhabdomyosarcoma cells [19,20]. On the other hand, the metabolic rewiring suffered by imatinib-resistant cells due to BCR-ABL1 overexpression has also been studied, thus being reported that these cells have enhanced glycolysis and decreased activity of the oxidative branch of pentose phosphate-pathway (PPP) [21]. Furthermore, CML cells harboring BCR-ABL1 mutations exhibit accumulation of TCA cycle intermediates, NADH/NAD^+^ increase, electron transport chain (ETC) alterations and low oxygen consumption [22]. However, to date, there has been less exploration about the metabolic rewiring associated with imatinib-resistant CML cells without BCR-ABL1 mutations and/or overexpression. Notably, although CML cells reside in a niche at very-low oxygen tension, all of the above-mentioned metabolic characterizations have been carried out under 21% oxygen conditions (normoxia).

In order to better understand the involvement of metabolic rewiring in the acquisition of BCR-ABL1-independent imatinib resistance, in this study we have performed a comprehensive metabolic comparison of imatinib-resistant (ImaR) cells with their respective syngeneic parental counterparts, both in normoxic and hypoxic conditions. We propose that a better knowledge of the metabolic requirements of KU812 ImaR cells will allow us to define potential metabolic vulnerabilities that could be pharmacologically assaulted towards more precise therapies for the management of TKIs-resistance.

## 2. Results

### 2.1. Resistance to Imatinib and Other TKIs Is Characterized by Alteration of Crucial Biological Processes

**Figure 1:**
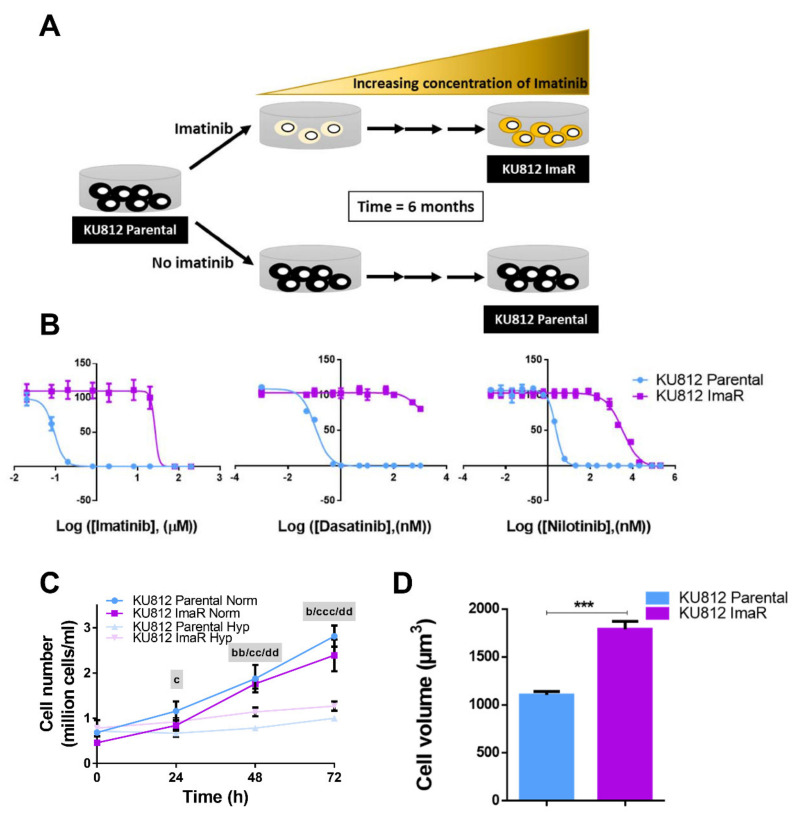
Phenotype, acquisition of TKIs resistance and changes in the principal biological processes of KU812 ImaR cells when compared with KU812 Parental cells. (**A**) Graphical description of the generation of an imatinib-resistant KU812 cell model (KU812 ImaR) under normoxia (Norm) by exposure of imatinib increasing concentrations. (**B**) Representative experiment (mean ± SD for *n* = 3 of *n* = 2 independent experiments) of the effect of three different clinically-relevant tyrosine kinase inhibitors (TKIs) on the cell viability of KU812 Parental and ImaR cell. Cells were incubated for 72 h with DMSO (vehicle control) or increasing concentrations of the corresponding inhibitor. Viability of the cells at each concentration was analyzed, and the half-maximal inhibitory concentrations (IC_50_) was determined. (**C**) Growth curve of KU812 parental and KU812 ImaR cells under normoxia and hypoxia (Hyp). The data represent mean ± SD for *n* = 3 of *n* = 2 independent experiments. Statistically significant differences were determined by two-tailed independent sample Welch’s *t*-test between KU812 ImaR and KU812 Parental in hypoxia (b), KU812 Parental in normoxia vs. hypoxia (c), or KU812 ImaR in normoxia vs. hypoxia (d) (*p* < 0.05 (b, c), *p* < 0.01 (bb, cc or dd), *p* < 0.001 (ccc). (**D**) Cell volume of KU812 P and KU812 ImaR cells under normoxia measured with the ScepterTM Handheld Automated Cell Counter. Statistically significant differences in all the panels was determined by two-tailed independent sample Student’s *t*-tests: *** *p* < 0.001.

In order to investigate the metabolic rewiring underlying BCR-ABL1-independent imatinib resistance in CML, an imatinib-resistant cell line (here referred as KU812 ImaR or TKI-resistant) was developed using KU812 cell line (here referred as KU812 parental or KU812 P), as depicted in Figure 1A and described in Material and Methods. KU812 ImaR cells enhanced their tolerance to imatinib by more than two orders of magnitude when compared to parental cells (drug resistance index (DRI) = 350). Furthermore, KU812 ImaR cells showed to also be resistant to the second generation TKIs dasatinib and nilotinib compared to the parental counterpart (DRIDasatinib > 8300 and DRINilotinib = 1280) (Figure 1B). To exclude the possibility that the reason for the development of resistance is only due to an altered expression of the BCR-ABL1 oncogene or even the occurrence of BCR-ABL1 gatekeeper mutation, we analyzed the resistant cells for the presence of the most common BCR-ABL1 mutations. No common mutations were detected in any of the relevant exons (including G250, Y253, E255, D276, F311, T315, F317, M351 and F359) (Figure S1), indicating that the resistance developed by our KU812 ImaR cells was not associated with a mutation in the kinase domain. Additionally, we were also able to rule out the reason for resistance formation due to the overexpression of the oncogene. Indeed, BCR protein was downregulated in the resistant cell line (4-fold down). We concluded that the metabolic rewiring is a feasible mechanism involved in the development of resistance and conducted a detailed metabolomic investigation of these cells. 

Differences in growth rate, cell cycle and morphology between KU812 P and ImaR cells were tested. There was no significant difference in cell proliferation between ImaR and parental cells under normoxia (Figure 1C). However, both cell lines showed a growth disadvantage under hypoxic conditions. Concerning cell cycle, KU812 ImaR cells showed a small but significant increase in S phase and a corresponding decrease in G0-G1 phase (Figure S2). Morphologically, KU812 ImaR cells were bigger (Parental cell diameter = 9.6 ± 0.85 µm and ImaR cell diameter = 10.4 ± 0.86 µm), and cell volume was 38 ± 4% larger in ImaR than in parental cells (Figure 1D), although protein content was equal for both cells.

**Figure 2:**
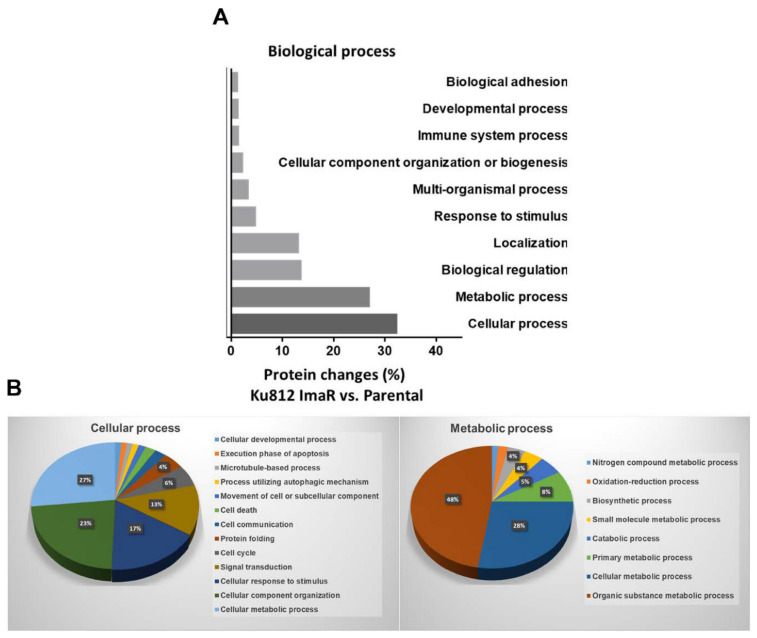
Pathway analysis of differentially expressed proteins identified by SILAC-based proteomics analysis of KU812 ImaR vs. KU812 P under normoxia. Only biological process whose contribution is >1% appear in the plot. Data are provided as mean ± SD of *n* = 2. (**A**) The bar chart shows biological processes enrichment analysis, based on the PANTHER gene ontology classification system. The up/down regulated proteins threshold for the analysis was defined as log_2_ fold change ≥ 0.58. (**B**) Relevant cellular and metabolic processes changes for KU812 ImaR compared to KU812 Parental cells.

To analyze in depth the effect of imatinib resistance acquisition we applied stable-isotope labelling by amino acids in cell culture (SILAC) to KU812 ImaR and parental cells in order to quantify the changes on protein level by mass spectrometry (MS) (Annex 1 in Supplementary Material). From a total of approximately 3200 identified proteins in the cell lines, 26.6% of them were upregulated and 26.0% were downregulated in the KU812 ImaR cells compared to the parental cells. The analysis of the biological processes in which these proteins are involved revealed that cellular processes (32.2%), considering any process that is carried out at the cellular level but not necessarily restricted to a single cell, and metabolic processes (26.8%) were the most altered in the acquisition of the resistant phenotype (Figure 2A). Moreover, a deeper analysis of the altered cellular processes showed that 24.2% of them also included cellular metabolic processes (Figure 2B). Thus, this analysis revealed that reprogramming of metabolism plays a key role in the acquisition of imatinib resistance of KU812 cells.

### 2.2. Development of Resistance to Imatinib Increased Glycolysis and Rewired Glucose to Pentose Phosphate Pathway, Glycogen Synthesis, and Serine-Glycine-One-Carbon Metabolism

**Figure 3:**
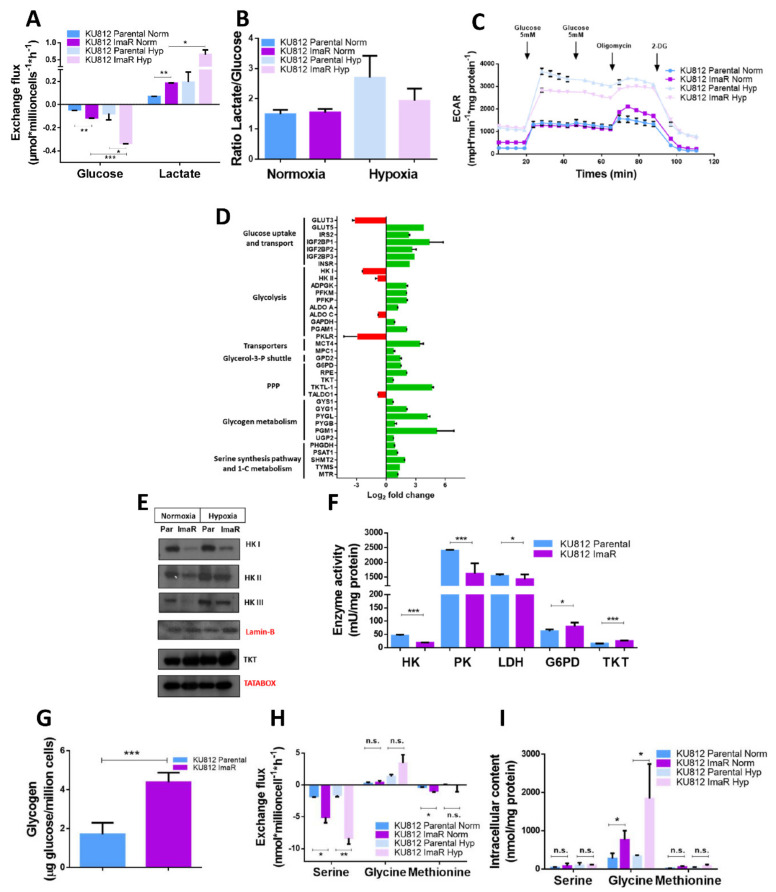
Effects of imatinib resistance on glycolysis, glycogen metabolism, pentose phosphate pathway and one-carbon metabolism. (**A**) Exchange fluxes of KU812 Parental (P) and imatinib-resistant (ImaR) cells. Glucose and lactate production rates were obtained under normoxia (Norm) and hypoxia (Hyp). Data (mean ± SD for *n* = 3 of *n* = 2 independent experiments) were normalized to cell number and incubation time. (**B**) Ratio lactate produced by glucose consumed using glucose and lactate flux rates under normoxia and hypoxia. Data are mean ± SD for *n* = 3 of *n* = 2 independent experiments. (**C**) Extracellular acidification rate (ECAR) measured under normoxia using a XF96 Extracellular Flux Analyzer during sequential injection of glucose, oligomycin and 2-deoxyglucose (2-DG) in KU812 Parental (P) and imatinib-resistant (ImaR) cells previously incubated under normoxic (Norm) and hypoxic (Hyp) conditions. Cells were first incubated in the absence of glucose. Data are mean ± SD for *n* = 8 replicates of three independent experiments. (**D**) Differences on the protein profiling of proteins associated with the glucose uptake, glucose metabolism and its rewiring to pathways such as glycerol-3-P shuttle, glycogen metabolism, pentose phosphate pathway (PPP), serine synthesis and 1-C metabolism in KU812 cells upon imatinib resistance. The protein expression profiling was obtained under normoxia using SILAC-based proteomic experiments. (**E**) Western blotting analysis of total protein fractions associated with glycolysis and PPP metabolism of KU812 Parental and ImaR cells under normoxia or hypoxia (1% O_2_). Data are mean ± SD for *n* = 3 of *n* = 2 independent experiments. (**F**) Hexokinase (HK), pyruvate kinase (PK), lactate dehydrogenase (LDH), glucose-6-phosphate dehydrogenase (G6PD) and transketolase (TKT) specific enzyme activities in normoxia. Data are mean ± SD for *n* = 3 of *n* = 2 independent experiments. (**G**) Glycogen levels in KU812 Parental and ImaR resistant cells in normoxia. The data are mean ± SD for *n* = 3 of *n* = 2 independent experiments. GC-MS was used to assess the glycogen content of parental (P) and ImaR cells. Measurement of the glycogen content was carried out using [U-^13^C-D7]-glucose as recovery standard and internal standard quantification procedures. Glucose from glycogen was corrected by millions of cells. (**H**) Representative experiment (mean ± SD for *n* = 3 replicates) of the serine, glycine and methionine consumption and production rates in KU812 Parental and ImaR cells measured by HPLC-MS/MS. (**I**) Intracellular concentrations of serine, glycine and methionine in KU812 Parental and ImaR cells. The data are mean ± SD for *n* = 3 replicates. Statistically significant differences in all the panels was determined by two-tailed independent sample Welch’s *t*-test when *n* < 3 and Student’s *t*-tests when *n* > 3: * *p* < 0.05, ** *p* < 0.01, *** *p* < 0.001, and *p* ≥ 0.05 (n.s. = non-significant differences). Abbreviations: ALDO, aldolase; GAPDH, glyceraldehyde-3-phosphate dehydrogenase; ADPGK, ADP-glucokinase; GLUT, glucose transporter; GPD2, glycerol-3-phosphate dehydrogenase; GYG, glycogenin; GYS, glycogen synthetase; IGF2BP, insulin like growth factor; INSR, insulin receptor; IRS2, insulin receptor substrate; MCT, monocarboxylate transporter; MPC, mitochondrial pyruvate carrier MTR, 5-methyltetrahydrofolate-homocysteine methyltransferase; PFKM, phosphofructokinase muscle isoform; PFKP, phosphofructokinase platelet isoform; PGAM, phosphoglycerate mutase; PGM, phosphoglucomutase; PHGDH, phosphoglycerate dehydrogenase; PPP, pentose phosphate pathway; PSAT, phosphoserine aminotransferase; PYG, glycogen phosphorylase; RPE, ribulose-5-phosphate-3-epimerase; SHMT2, serine hydroxymethyltransferase; TALDO1, transaldolase 1; TKTL1, transketolase like 1; TYMS, thymidylate synthetase; and UGP2, UDP-glucose pyrophosphorylase 2.

The observation that around 20% of the proteins up/down regulated were associated with metabolic processes prompted us to further analyze the rewiring of the main pathways of central carbon metabolism underlying acquired BCR-ABL1-independent imatinib resistance. Since glycolysis was shown to be affected by mutation-dependent acquisition of imatinib-resistance, we aimed to evaluate whether mutation-independent resistance acquisition is also accompanied by an altered glucose metabolism. To that end, we measured the glucose consumption and lactate production rates in KU812 P and ImaR cells. Both rates were higher in ImaR than in parental cells, both in normoxia (60% higher) and in hypoxia (70% higher) (Figure 3A). Moreover, the ratio between lactate production and glucose consumption was the same for ImaR and parental cells both in normoxic and hypoxic conditions, indicating that the increase undergone by both rates in KU812 ImaR cells was proportional (Figure 3B). However, the increase in lactate production observed in KU812 ImaR cells was not accompanied by extracellular acidification (ECAR) neither under normoxia nor under hypoxia (Figure 3C and Figure S3A). These results suggest that KU812 ImaR cells may have enhanced other processes that may utilize the excess of H^+^ formed with lactate during anaerobic glycolysis. Indeed, the downregulation of the CA1 and CA2 protein expressions (13.59 and 15.65-fold down, respectively) together with the upregulation of SLC4A7 transporter (3.45-fold) again confirmed a decreased contribution of CAs to the ECAR value and a compensatory mechanism of intracellular bicarbonate balance through extracellular HCO_3_^−^ import in KU812 ImaR cells (Figure S3B). In parallel to ECAR measurements, the Crabtree effect was analyzed by measuring the oxygen consumption rate (OCR) changes after glucose addition under normoxia, in KU812 P and ImaR cells pre-incubated under normoxic or hypoxic conditions, in order to analyze the dependence on glucose as a source of energy. Results showed that the OCR rate decreased dramatically after glucose addition in KU812 P cells pre-incubated under normoxic conditions (Figure S3C). On the contrary, the basal OCR in the absence of glucose was maintained after glucose addition in KU812 ImaR cells pre-incubated under normoxic conditions, indicating a total absence of the Crabtree effect in the KU812 ImaR cells. After pre-incubation of the cells in hypoxic conditions, the Crabtree effect was also significantly lower in KU812 ImaR when compared to KU812 P cells. These results suggest that resistant cells have an increased dependency on OXPHOS and are therefore potentially more vulnerable to drugs targeting mitochondria.

To further analyze differences in glycolysis between KU812 P and ImaR cells, protein levels were analyzed by MS, as described before, and by Western blotting analysis. The expression of most of the proteins involved in glycolysis was upregulated in KU812 ImaR cells, although hexokinase (HK) isoforms and the liver isoform of pyruvate kinase (PK) (PKLR) were downregulated (Figure 3D). In order to confirm the above-observed lower expression of the different HK isoforms, we next performed Western blot analysis to validate these proteomics results. HKI, II and III showed a reduced expression in KU812 ImaR relative to KU812 P cells (Figure 3E). Moreover, HK and PK enzyme activities were significantly reduced in KU812 ImaR compared to KU812 P cells, whereas lactate dehydrogenase (LDH) activity decreased less than 10% in accordance with the fact that LDH protein levels did not change significantly (Figure 3F). 

The fact that the increase in glucose consumption and lactate production in KU812 ImaR cells was proportional, in addition to the observed increase in lactate flux, leads to the hypothesis that other branches of glucose metabolism such as oxidative and non-oxidative PPP, glycogen metabolism, serine-glycine-1C (SGOC) metabolism, glycerol metabolism and pyruvate/lactate transport to mitochondria could be enhanced. Glucose-6-phosphate dehydrogenase (G6PD) (the rate limiting enzyme of the oxidative PPP) and transketolase (TKT) (the rate limiting enzyme of the non-oxidative PPP) were upregulated in KU812 ImaR cells both in normoxia and in hypoxia (Figure 3D,E). Consistently, their enzyme activities were 30 and 63% higher, respectively, in ImaR than in parental cells in normoxia (Figure 3F). It is noteworthy to mention that KU812 ImaR cells showed a significant up-regulation of the transketolase like-1 isoform (TKTL1) when compared to parental cells, indicating that TKTL1 upregulation could be an important enzyme in the acquisition of the resistant phenotype. 

In addition, we observed a 2.6-fold increase in glycogen content in KU812 ImaR cells compared to parental cells (Figure 3G). Consistently, phosphoglucomutase 1 (PGM1), the enzyme which indirectly regulates glycogen and pentose-phosphate synthesis by fine tuning of the glucose-1-P and glucose-6-P balance, was strongly upregulated (FC = 34.15) in KU812 ImaR cells (Figure 3D). Moreover, we also observed that glycogen phosphorylase isoenzymes (PYGL and PYGB), responsible for glycogen degradation and important for the optimal function of the PPP [23], were upregulated in KU812 ImaR cells. Altogether, these results suggest that the enhanced glycogen synthesis/degradation cycle is necessary to simultaneously sustain glycogen accumulation and to satisfy PPP activity in the KU812 ImaR cells. 

Furthermore, as PK activity is decreased in KU812 ImaR cells, we hypothesized that resistant cells may use glycolysis intermediates, such as 3-P-glycerate, to fuel serine synthesis and one-carbon metabolic pathways. These pathways play a crucial role in purine and DNA synthesis and in methylation processes such as DNA methylation and protein post-translational modifications. Upregulation of PHGDH and PSAT (key players of SSP), and SHMT2, TYMS and MTR (key players of 1-C metabolism) in KU812 ImaR relative to KU812 P cells were observed (Figure 3D). Moreover, the higher flux through these two pathways is consistent with the higher serine and methionine consumption and glycine intracellular content observed in KU812 ImaR cells both in normoxia and in hypoxia (Figure 3H–I). These results indicate that the SSP and 1C-metabolism are also crucial pathways influencing glucose metabolism of KU812 ImaR cells.

To further explore the glucose metabolism network at the level of 3-carbon molecules, we analyzed the proteomic changes associated with the glycerol metabolism and pyruvate/lactate mitochondrial transporters. Results show that glycerol-3-phosphate dehydrogenase (GPD2), the mitochondrial pyruvate carrier MPC1, as well as the monocarboxylate carriers MCT4 (SLC16A3) were upregulated in KU812 ImaR cells (Figure 3D). It is worth mentioning that MCT4 has been described to be localized within the plasmatic and mitochondrial membranes and to be able to import/export lactate/pyruvate between mitochondria and cytosol in symport with a H^+^ [24]. These changes in pyruvate and monocarboxylate carrier are indicative of an increased flux of pyruvate/lactate into the mitochondria, further suggesting a stronger dependence on pyruvate to support mitochondrial respiration. Moreover, the increase in GPD2 observed in KU812 ImaR cells indicates a stronger dependence on glycerol-3-phosphate (GP) shuttle, thus additionally suggesting an altered mitochondrial respiration capacity.

### 2.3. Development of Resistance to Imatinib Rewired Glutamine Metabolism to Increase Proline Synthesis and Enhanced Self-Protection Mechanisms through Glutathione Metabolism

**Figure 4:**
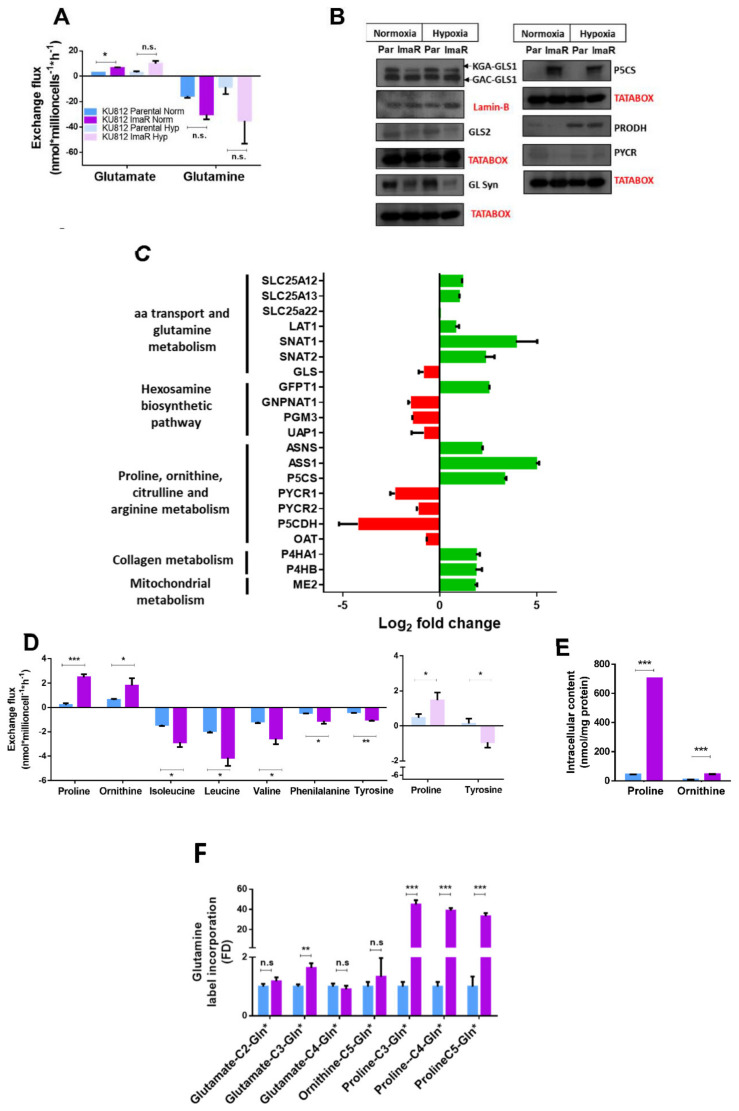
Imatinib resistance results in the rewiring of glutamine/glutamate metabolism to proline, collagen and glutathione metabolism. (**A**) Extracellular metabolic fluxes of KU812 Parental and imatinib resistant (ImaR) cells under normoxia (Norm) and hypoxia (Hyp). Data are mean ± SD for *n* = 3 of *n* = 2 independent experiments. Glutamine consumption and glutamate production were obtained under normoxia. Data were normalized to cell number and incubation time. (**B**) Western blotting analysis of total protein fractions related to glutamine and proline metabolism of KU812 Parental and ImaR cells under normoxia or hypoxia. Data are mean ± SD for *n* = 3 of *n* = 2 independent experiments. Kidney-type glutaminase 1 (KGA-GLS1), glutaminase C (GAC-GLS1), glutaminase 2 (GLS2), and glutamine synthetase (GS) (**C**) Protein profile of the rewiring of glutamine metabolism in KU812 imatinib-resistant when compared to KU812 parental cells (mean ± SD for *n* = 2 replicates). Log2 fold change values were calculated and represented by green color = protein upregulation; and red = protein downregulation. (**D**) Representative experiment of the amino acids consumption and production rates (mean ± SD for *n* = 3 replicates) significantly different between KU812 Parental and ImaR cells under normoxia and hypoxia. (**E**) Intracellular concentrations of amino acids significantly different between in KU812 Parental and ImaR cells under normoxia. The data are mean ± SD for *n* = 3 replicates. (**F**) Fold difference value (mean ± SD for *n* = 3 replicates) in ^13^C Glutamine label incorporation in some metabolites of the KU812 ImaR cells compared to KU812 Parental cells in normoxia. Statistically significant differences in all the panels was determined by two-tailed independent sample Welch’s *t*-test when *n* < 3 and Student’s *t*-tests when *n* > 3: * *p* < 0.05, ** *p* < 0.01, *** *p* < 0.001, and *p* ≥ 0.05 (n.s. = non-significant differences). Abbreviations: ASNS, aspargine synthetase; ASS1, arginosuccinate synthase; GFPT1, Glutamine-Fructose-6-Phosphate Transaminase 1; GNPNAT1, Glucosamine-Phosphate N-Acetyltransferase 1; OAT, ornithine aminotransferase; P4H, prolyl 4-hydroxylase; P5CDH, delta-1-pyrroline-5-carboxylate dehydrogenase; P5CS, delta-1-pyrroline-5-carboxylate synthase; PGM, phosphoglucomutase; PYCR, pyrroline-5-carboxylate reductase; ME, malic enzyme; SLC, solute carrier; SNAT, system N amino acid transporter; and UAP1, UDP-N-Acetylglucosamine Pyrophosphorylase 1.

The above results show that KU812 cells increased glucose consumption during the formation of imatinib resistance to operate other metabolic pathways besides glycolysis. Therefore, we next investigated differences in glutamine metabolism in the course of resistance development. The net glutamine consumption and glutamate production were detected to be higher in ImaR than in parental cells, although this increase was only significant in glutamate production in normoxia (Figure 4A). To elucidate whether the increase in glutamine consumption and glutamate production observed in KU812 ImaR cells was due to an increase in the glutaminase (GLS) isoenzymes or a decrease in glutamine synthetase (GS) (the main enzymes involved in glutaminolysis and glutamine synthesis, respectively), the expression of these proteins was quantified by Western blot analysis and by protein profile (SILAC) analysis. Overall, KU812 ImaR cells showed lower levels of GLS isoenzymes (GLS1 and GLS2) in the Western blot results, and a slight downregulation (1.69-fold down) of the total protein concentration. Furthermore, the increase in glutamine consumption observed in KU812 ImaR cells was consistent with the dramatic increase observed at protein level in the two main glutamine transporters, SLC38A1 (SNAT1) (15.11-fold up) and SLC38A2 (SNAT2) (5.00-fold up). Other amino acids transporters such as LAT1 were also upregulated (1.73-fold up) in KU812 ImaR cells, indicating that these cells not only have a stronger dependence on glutamine but also on other amino acid metabolic pathways (Figure 4B,C). All together, these results demonstrate that the observed changes in the glutamine consumed, and the glutamate produced in KU812 ImaR cells are not due to an increase in glutaminolysis despite the fact that glutamine import is upregulated in KU812 ImaR cells. 

Besides glutamine and glutamate, we also analyzed the exchange flux rates of additional amino acids and the intracellular content of all the amino acids in both cell lines (Tables S1–S4). KU812 ImaR cells consumed more branched-chain amino acids (BCAAs), phenylalanine and tyrosine, and produced more proline (13-fold increase) and more ornithine (3-fold increase), than parental cells in normoxia, while under hypoxic conditions they only consumed more tyrosine and produced more proline (3-fold increase) than parental cells (Figure 4D). Regarding the amino acids intracellular content, KU812 ImaR cells had 16 times more proline and seven times more ornithine than parental cells in normoxia (Figure 4E). 

Considering that both proline and ornithine can be synthesized from glutamate through glutamate-γ-semialdehyde (GSA) synthesis, but ornithine can also be synthesized from other carbon sources such as arginine, we next cultured KU812 P and ImaR cells in the presence of either uniformly ^13^C labelled [U-^13^C]-glucose or [U-^13^C]-glutamine. Subsequently, we analyzed the incorporation of ^13^C into glutamate, proline and ornithine metabolites by NMR (Figures S4 and S5). ^13^C incorporation coming from [U-^13^C]-glucose was neither observed in proline nor in ornithine in KU812 ImaR cells (Figure S4). However, ^13^C incorporation in the carbons C3, C4 and C5 of proline was 39-fold higher in KU812 ImaR than in KU812 P cells when [U-^13^C]-glutamine was used, although ^13^C incorporation in C2 and C4-glutamate and in C5-ornithine was the same in KU812 ImaR and in P cells (Figure 4F). These results suggest that the higher proline synthesis observed in KU812 ImaR cells was directly linked to glutamine metabolism, while the mechanism responsible for the higher ornithine synthesis in KU812 ImaR cells was independent of glutamine metabolism. 

To better elucidate the metabolic role of proline in KU812 ImaR cells, the protein profile of proteins associated with proline metabolism and amino acids closely related to proline metabolism was revisited. The rate limiting enzyme of proline and ornithine synthesis, pyrroline-5-carboxylate synthase (P5CS, also called ALDH18A1), was highly upregulated in KU812 ImaR cells (9.91-fold up). This upregulation was accompanied by the downregulation of the enzyme that catalyzes the reverse reaction, the delta-1-pyrroline-5-carboxylate dehydrogenase (P5CDH or ALDH4A1) (17.63-fold down) and of the two isoforms of pyrroline-5-carboxylate reductase (PYCR) 1 and 2 (4.70-fold up and 2.04-fold down). Conversely, proline dehydrogenase (PRODH) and delta-1-pyrroline-5-carboxylate dehydrogenase (P5CDH), both enzymes responsible for the proline oxidation, were downregulated in KU812 ImaR cells in normoxia. It is known that proline and hydroxyproline are required to maintain collagen stability [25]. Consistently, we observed that enzymes responsible for collagen synthesis, prolyl 4-hydroxylases (P4H), were also upregulated in KU812 ImaR cells compared to the parental ones (Figure 4B,C). These results were consistent with an increased production of proline by KU812 ImaR cells. 

Furthermore, we examined the protein expression of the mitochondrial amino acid transporters by using protein profile analysis. SLC25A12 and SLC25A13 transporters, which are involved in the exchange of mitochondrial aspartate for cytosolic glutamate across the inner mitochondrial membrane, showed an upregulation in KU812 ImaR compared to KU812 P cells (Figure 4C). Consistent with the observed increase in ASNS, the upregulation of these transporters in KU812 ImaR cells indicates an enhanced aspartate/glutamate transport, which can be driven by an increased demand for cytosolic aspartate for asparagine synthesis. Moreover, KU812 ImaR cells also exhibited a dramatic upregulation of argininosuccinate synthase 1 (ASS1) (31.07-fold up), the rate-limiting enzyme of arginine synthesis, which catalyzes the synthesis of arginosuccinate from citrulline and aspartate (Figure 4C). This fact, together with the observed upregulation of the SLC25A12 and SLC25A13 transporters, suggests an increased requirement of aspartate in the cytosol to serve the need of aspartate-dependent biosynthetic reactions in KU812 ImaR cells. On the other hand, the upregulation of ASS1 observed in KU812 ImaR cells also suggests an enhancement of the pathway producing arginine and fumarate through the sequential reactions of ASS1 and argininosuccinate lyase (ASL). It is also worth mentioning that the mitochondrial malic enzyme (ME2), which converts malate into pyruvate within the mitochondria, was also upregulated (3.46-fold up) in KU812 ImaR compared to KU812 P cells.

**Figure 5:**
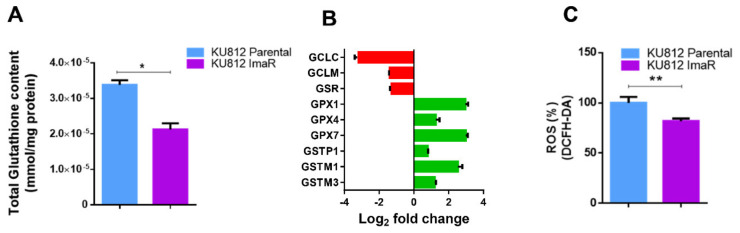
Differences of glutathione metabolism between KU812 imatinib-resistant and KU812 Parental cells. (**A**) Total glutathione levels in KU812 Parental and imatinib-resistant (ImaR) cells in normoxia. Data are mean ± SD for *n* = 3 replicates. (**B**) The protein expression profiling of proteins associated with glutathione metabolism in KU812 ImaR and Parental cells under normoxia was obtained using SILAC-based proteomic experiments. Log2 fold change values were calculated and represented by green color = protein upregulation; and red = protein downregulation. (**C**) Intracellular reactive oxygen species (ROS) levels in KU812 Parental and ImaR cells in normoxia. Data are mean ± SD for *n* = 3 replicates. Statistically significant differences in all the panels were determined by two-tailed independent sample Welch’s *t*-test when n < 3 and Student’s *t*-tests when n > 3: * *p* < 0.05, ** *p* < 0.01. Abbreviations: GCLC, glutamate-cysteine ligase catalytic subunit, GCLM, glutamate-cysteine ligase modifier subunit; GPX, glutathione peroxidase; GSR, glutathione-disulfide reductase; and GST, glutathione-S transferase.

Another metabolite directly linked to glutamate (and consequently to glutamine) metabolism and closely associated with drug resistance is glutathione [26,27]. Thus, we investigated whether glutamine metabolism was rewired to glutathione synthesis in KU812 ImaR cells. The results show that total glutathione levels as well as ^13^C incorporation (coming from [U-^13^C]-glutamine) into reduced glutathione (GSH) were lower in KU812 ImaR than in KU812 P cells (Figure 5A and Figure S5). Protein levels of glutamate–cysteine ligase (GCL), both the catalytic and regulatory subunits, were reduced in KU812 ImaR (9.04- and 2.62-fold down, respectively) compared to KU812 P cells (Figure 5B). Altogether, these results indicate that the de novo glutathione synthesis does not contribute to the increase in glutamine consumption observed in KU812 ImaR cells. Regarding the protein levels of other proteins associated with glutathione metabolism, glutathione peroxidases (GPX1, GPX4 and GPX7) as well as glutathione-S-transferases including (GSTP1, GSTM1, and GSTM3), were strongly upregulated in KU812 ImaR cells (Figure 5B). Moreover, reactive oxygen species (ROS) levels were further analyzed to verify our hypothesis. Consistently, a decrease of 18% in the ROS levels of KU812 ImaR was determined when compared to KU812 P cells (Figure 5C). Therefore, these results highlight an important role of glutathione in the detoxification reactions in KU812 ImaR cells despite the reduced de novo synthesis of glutathione.

### 2.4. Development of Resistance to Imatinib Implied A Reorganisation of Mitochondrial Metabolism and Electron Transport Chain Activities to Increase Mitochondrial Respiration

**Figure 6:**
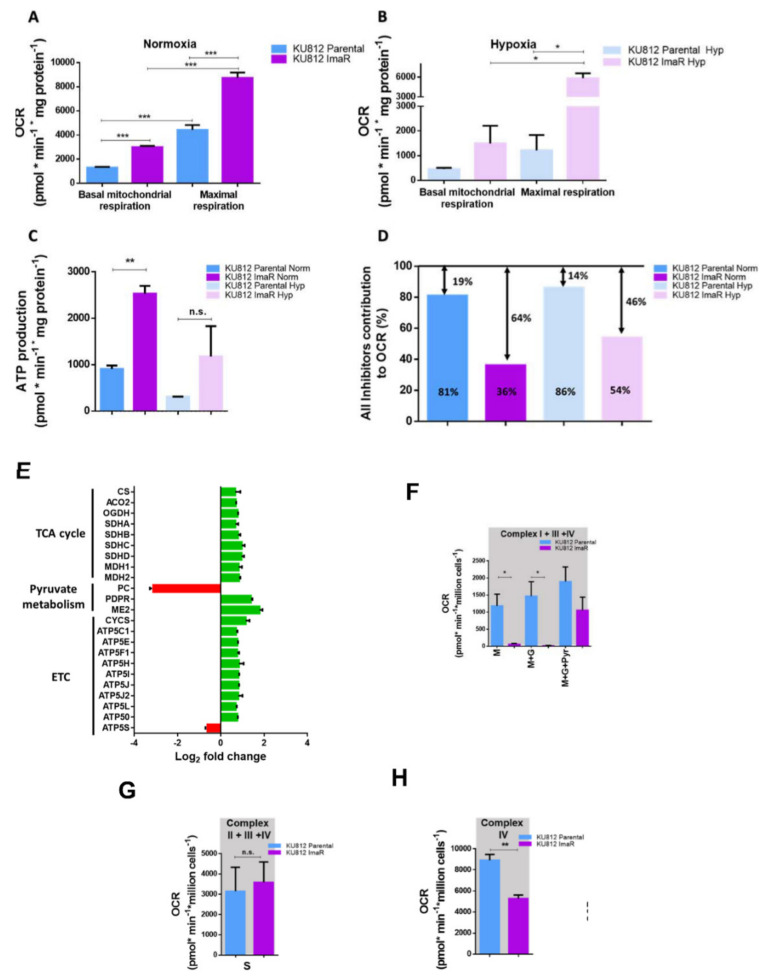
Characterization of mitochondrial respiration in KU812 imatinib-resistant vs. KU812 Parental cells. (**A**–**C**) Oxygen consumption rate (OCR) values were measured under normoxia during sequential injection of oligomycin, CCCP, and Rot+Ama in KU812 Parental and imatinib-resistant (ImaR) cells previously incubated under normoxic and hypoxic conditions. Basal and maximal respiration of cells incubated under normoxic (panel **A**) and hypoxic (panel **B**) conditions were calculated. ATP production-associated respiration of KU812 Parental vs. ImaR cells previously incubated under normoxic and hypoxic conditions (panel **C**) were calculated as explained in material and methods. (**D**) Fatty acid, glutamine and glucose/pyruvate contributions to mitochondrial respiration in KU812 Parental and ImaR cells previously incubated under normoxic and hypoxic conditions were determined by measuring OCR under normoxia during sequential injections of Etomoxir, BPTES, UK5099 and Oligomycin inhibitors. Data were normalized by protein. Data are provided as mean ± SD of *n* = 3. (**E**) Protein expression profiling of proteins associated with mitochondrial metabolism, pyruvate metabolism and electron transport chain. Log2 fold change values were calculated and represented by green color = protein up-regulation; and red = protein down-regulation. (mean ± SD for *n* = 2 replicates. (**F**–**H**) Oxygen consumption rate (OCR; expressed as picomoles (pmol) per minute (min) per million of cells (Mill)) measurements of permeabilized KU812 Parental and ImaR cells was determined at 37 °C in 2 mL glass chambers using a two-channel, high-resolution Oxygraph respirometer in normoxia as indicated in material and methods. OCR was measured after sequential injections of substrates and inhibitors with an addition of 5 mM ADP together with the first substrate added. Data (mean ± SD for *n* = 3 of *n* = 3 independent experiments) was analyzed using DatLab7 software. OCRs). The oxygen flow in these states was corrected by the subtraction of non-mitochondrial respiration (ROX), which was obtained after the addition of antimycin (Ama). For (**F**) (approach to study of complex I): 2 mM malate (M), 10 mM glutamate (G), and 5 mM pyruvate (P). For (**G**) (approach to study complex II): 10 mM succinate (S). For (**H**) (approach to study complex IV activity): 5 mM Ascorbate (As) plus 0.5 mM N,N,N′,N′-tetramethyl-p-phenylendiamine dihydrochloride (TMPD). Statistically significant differences in all the panels were determined by two-tailed independent sample Student’s *t*-tests: * *p* < 0.05, ** *p* < 0.01, *** *p* < 0.001, and *p* ≥ 0.05 (n.s. = non-significant differences). Abbreviations: ACO, aconitase; ATP5, ATP synthase; CS, citrate synthase; CYCS, cytochrome c; OGDH, oxoglutarate dehydrogenase; PC, pyruvate carboxylase; PDPR, pyruvate dehydrogenase phosphatase regulatory subunit; MDH, malate dehydrogenase; ME, malic enzyme; and SDH, succinate dehydrogenase.

We next investigated mitochondrial respiration differences between ImaR and parental cells in normoxia and hypoxia. KU812 ImaR cells exhibited higher basal and maximal respiration capacity compared to KU812 P cells, both after normoxic and hypoxic incubation conditions. Indeed, ATP production was also increased upon imatinib resistance acquisition under normoxic incubation conditions (Figure 6A–C). We also assessed the contribution of glutamine, pyruvate (from glucose or other carbon sources) and fatty acid oxidation to mitochondrial respiration in both cell lines under normoxia and hypoxia. The results show that these substrates entailed more than 80% of oxygen consumption in parental cells both under normoxia and hypoxia, but they contributed to a much lower extent to oxygen consumption in KU812 ImaR cells (36% in normoxia and 54% in hypoxia) (Figure 6D), even though resistant cells exhibited higher mitochondrial respiration than parental cells. Thus, KU812 ImaR cells can utilize other sources to perform mitochondrial respiration. 

To complete the characterization of the mitochondrial metabolism, we further looked at the protein profile of proteins involved in the mitochondrial metabolism. Results exhibited a higher expression of several proteins of the TCA cycle including citrate synthase (CS), aconitase 2 (ACO2), oxoglutarate dehydrogenase (OGDH), succinate dehydrogenase (SDH), and malate dehydrogenase (MDH) in KU812 ImaR compared to KU812 P cells (Figure 6E). Furthermore, upregulation of pyruvate dehydrogenase (PDH) activation by pyruvate dehydrogenase phosphate (PDP) and downregulation of pyruvate carboxylase (PC) shown in MS data, indicate that pyruvate entrance into TCA cycle was fueled to be metabolized to Acetyl-CoA through PDH. In addition, ImaR cells showed a 3.46-fold increase in NAD-dependent mitochondrial isoform of malic enzyme (ME2) that would redirect mitochondrial malate to form pyruvate.

The mitochondrial network is characterized by the final ATP production through the coupled integration of the electron transport chain (ETC) with oxidative phosphorylation. In our study, we have demonstrated that KU812 ImaR exhibited a higher protein expression of the SDH (complex II), the cytochrome c (CYCS), and the ATP synthase (ATP5) subunits (complex V) including ATP5C1, ATP5E, ATP5F1, ATP5H, ATP5I, ATP5J, ATP5J2, ATP5L, and ATP50, when compared to KU812 P cells. On the other hand, non-significant differences were observed in catalytic protein subunits associated with complex I, III and IV between KU812 P and ImaR cells (Figure 6E).

In light of the upregulation of key proteins for ETC function, we next investigated the differences in ETC complex activities associated with imatinib resistance acquisition by using permeabilized cells and measuring OCR using the Oroboros Oxygraph-2k respirometer after the injection of different inhibitors and substrates. Complex I activity was significantly lower in KU812 ImaR relative to KU812 P cells when complex I substrates malate and glutamate were only added. Moreover, complex I activity only increased in KU812 ImaR cells until pyruvate was further added. This result suggests that the complex I activity of KU812 ImaR cells turned out to be dependent on pyruvate, unlike in KU812 P cells, whose mitochondria were able to consume O_2_ even with only malate as substrate (Figure 6F). Regarding the complex II activity, KU812 ImaR cells exhibited the same activity as KU812 P cells when succinate was added as a substrate (Figure 6G). Finally, a lower maximal activity of complex IV was observed in KU812 ImaR cells when compared to KU812 P cells (Figure 6H). Acute drug exposure to imatinib did not induce complex I pyruvate dependency and neither did it reduce complex IV activity in KU812 P cells. Moreover, no significant differences were found regarding complex II activity (Figure S6). Thus, we confirmed that the mitochondrial phenotype exhibited in this study by KU812 ImaR cells was due to the imatinib resistance acquisition. These results point out that the available complex IV in KU812 ImaR cells has enough capacity to absorb the electrons coming from previous complexes and that the control of the ETC relies on the previous steps.

**Figure 7:**
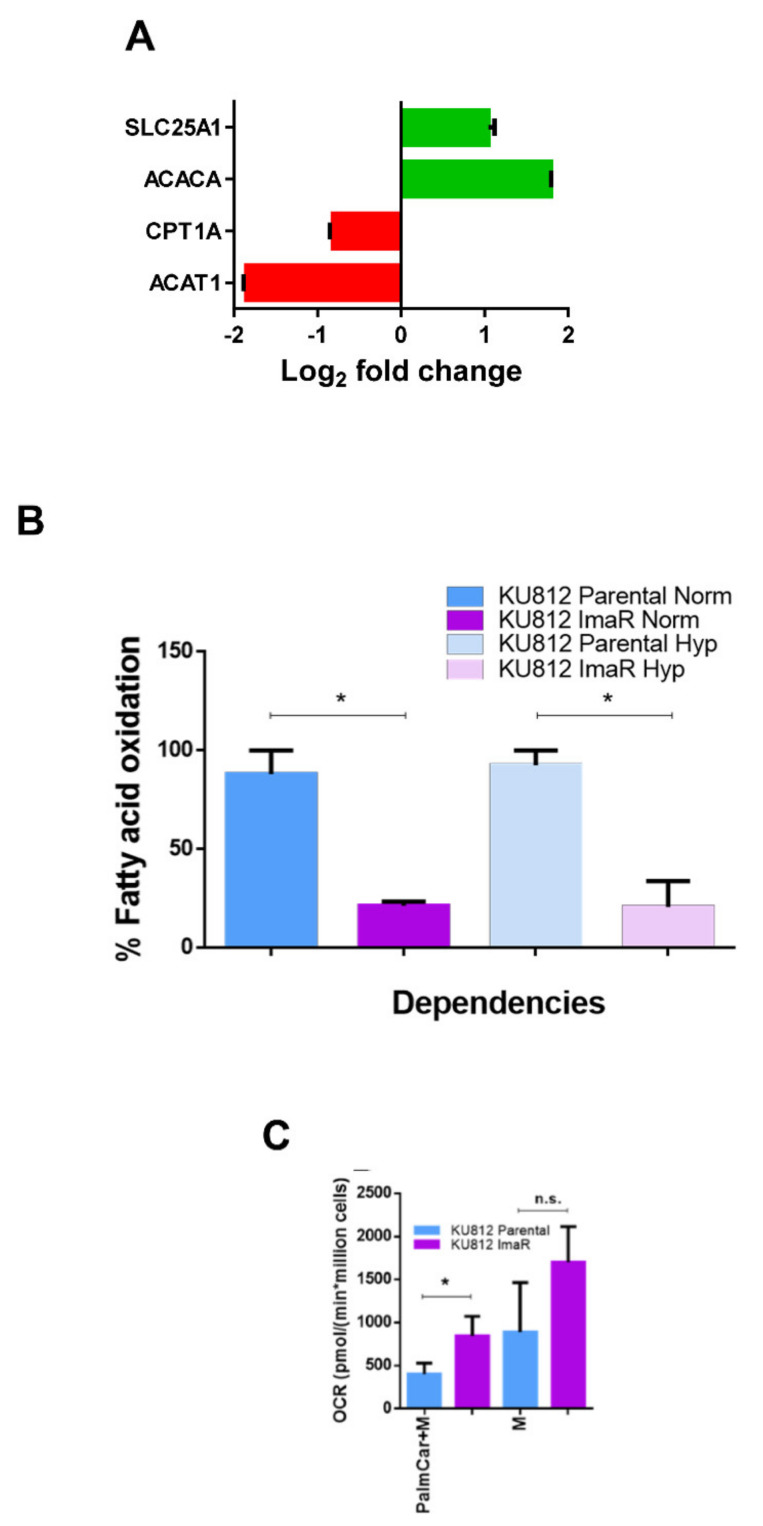
Acquisition of imatinib resistance alters the fatty acid metabolism of KU812 cells. (**A**) Protein profiling differences of KU812 imatinib-resistant (ImaR) cells when compared to KU812 Parental cells regarding fatty acid metabolism. The protein expression profiling was obtained using SILAC-based proteomic experiments. Log2 fold change values were calculated and represented by green color = protein upregulation; and red = protein downregulation. Data are provided as mean ± SD of *n* = 2. (**B**) Fatty acid contribution to mitochondrial respiration using Mitofuel Flex Test results of KU812 Parental and ImaR cells under normoxia (Norm) and hypoxia (Hyp). All the results were calculated as explained in Materials and Methods. The figure shows the mean ± SD for *n* = 3. (**C**) Oxygen consumption rate (OCR) measurements of permeabilized KU812 Parental and ImaR cells in normoxia when adding fatty acids (PalmCar, palmitoyl-DL-carnitine-HCl) plus 0.1 mM malate (M). Significance was determined by two-tailed independent sample Student’s *t*-test. Statistically significant differences between KU812 Parental and KU812 ImaR cells were indicated as * *p* < 0.05 and *p* ≥ 0.05 (n.s. = non-significant differences). Abbreviations: ACACA, acetyl-CoA carboxylase alpha; ACAT, acetyl-CoA acyltransferase; CPT1, carnitine palmitoyltransferase 1; and SLC, solute carrier.

Regarding the beta-oxidation, we observed that the protein levels of almost all the enzymes involved in this pathway were enhanced in KU812 ImaR cells, except for carnitine palmitoyl transferase 1 (CPT1) and acetyl-CoA acetyltransferase 1A (ACAT1) (1.76 and 3.61-fold down, respectively) (Figure 7A). In fact, we observed that KU812 ImaR cells were much less dependent on beta-oxidation than parental cells (Figure 7B). In order to confirm that beta-oxidation machinery was enhanced inside mitochondria and this pathway was controlled by CPT1 levels, we checked the OCR when palmitoyl-CoA carnitine was administered to permeabilized cells (Figure 7C). Results showed that mitochondria from KU812 ImaR cells had higher OCR than mitochondria from parental cells when CPT1 is bypassed, thus confirming that the lower dependency on beta-oxidation in KU812 ImaR cells is mainly dependent on CPT1 levels and not on ACAT1.

**Figure 8:**
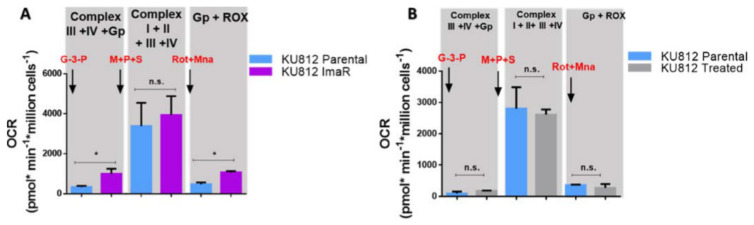
Contribution of glycerol-3-phosphate shuttle to the mitochondrial respiration of KU812 imatinib-treated or KU812 imatinib-resistant vs. KU812 Parental cells. (**A**,**B**) OCR measurements of KU812 imatinib-resistant (ImaR) vs. KU812 Parental (P) (Panel **A**), and KU812 Treated (KU812 P cells treated with 80 nM imatinib for 72 h under normoxic conditions) vs. KU812 P cells (panel **B**) determined under normoxia by Oroboros Oxygraph-2k respirometer. OCR were measured after sequential injections of 10 mM Gp + 5 mM ADP, 10 mM succinate + 5 mM pyruvate + 2 mM malate, 5 mM malonic acid, and 0.5 µM rotenone + 2.5 µM antimycin. Data were normalized by cell number. Data are provided as mean ± SD of *n* = 3 and significance was determined by two-tailed independent sample Student’s *t*-test. Statistically significant differences between KU812 Parental and KU812 ImaR cells were indicated as * *p* < 0.05 and *p* ≥ 0.05 (n.s. = non-significant differences). Abbreviations: G-3-P, glycerol-3-phosphate; P, pyruvate; M, malate; Mna, malonic acid; Rot, rotenone; and S, succinate.

In trying to understand how resistant cells enhanced their mitochondrial respiration compared to parental cells, even though complex IV was less active in ImaR than in parental cells, other pathways that could fuel mitochondrial respiration were examined. The mitochondrial isoform of glycerol-3-phosphate dehydrogenase (GPD2) converts glycerol-3-P (GP) to dihydroxyacetone-P, thus transferring 2 e^−^ and 2 H^+^ onto FAD, forming FADH_2_ in the mitochondrial intermembrane [28]. In the next step, a ubiquinone molecule in the core of the inner membrane collects the two 2 e^−^ and 2 H^+^, thereby reducing ubiquinone to ubiquinol with concurrent oxidation of FADH_2_ to FAD. This mechanism is known as GP shuttle. Next, ubiquinol transfers electrons to complex III allowing mitochondria to produce ATP independently of complex I and II activities (Figure S7). As noticed during protein expression profiling analysis, KU812 ImaR cells upregulated GPD2 (Figure 3D). Moreover, and as mentioned above, complex III (CYCS protein) was also upregulated in KU812 ImaR cells. To ensure that upregulation of GPD2 and CYCS induced mitochondrial respiration in resistant cells, OCR rates were measured in both KU812 ImaR and P cells when GP was added as a substrate. As shown in Figure 8A, KU812 ImaR cells exhibited an increase in mitochondrial respiration when GP was added, confirming that GPD2 contributed to mitochondrial respiration in imatinib resistant cells. The acute drug exposure to imatinib was not able to exhibit mitochondrial respiration due to GPD2 activity (Figure 8B), thus confirming that the mitochondrial phenotype exhibited by KU812 ImaR cells was due to the imatinib resistance phenotype.

### 2.5. Drug Combination, Targeting Distinct Metabolic Pathways, Showed to Be the Selected Way to Overcome TKI Resistance

All the above results highlighted different metabolic pathways as possible targets that could be inhibited to test whether the pharmacologic inhibition of metabolic processes shows to be important for TKI resistance. In this spirit, we treated ImaR and parental cells with a GPX4 inhibitor (RSL3), three different PYG inhibitors (1,4-dideoxy-1,4-imino-d-arabinitol (DAB), CP-320626 and CP-91149), a DHFR inhibitor (Methotrexate), a DHFR-GARFT-TYMS inhibitor (Pemetrexed), a SHMT inhibitor (Shin2), a G6PD inhibitor (Dehydroepiandrosterone (DHEA)), and a GSTP1 inhibitor (Ezatiostat), to inhibit GSH synthesis, glycogenolysis, one-carbon metabolism, oxidative-PPP, and S-glutathionylation, respectively. Results showed that IC_50_s obtained for Methotrexate and Pemetrexed and RSL-3 were in the range of nanomolar for both parental and TKI-resistant cells unveiling one-carbon metabolism and glutathione metabolism as putative targets to treat CML and to counteract TKI-resistance. Furthermore, the IC_50_ values obtained for the inhibitors targeting S- glutathionylation, PPP and glycogenolysis were in micromolar range (Table 1 and Figure S8).

The fact that we observed a similar effect on KU812 P and ImaR cells open new avenues to be explored in combined therapies to manage TKI-resistance. Thus, taking into account that both KU812 P and ImaR cell lines were highly sensitive to the inhibition of glutathione metabolism, and that KU812 ImaR cells display an enhanced mitochondrial respiration, we reasoned that ImaR will be more sensitive to Doxorubicin, a drug extensively used in the management of acute myelogenous leukemia (AML) [29] that induces oxidative stress and disrupts mitochondrial oxidative phosphorylation [30]. The fact that we obtained a 13-fold lower IC_50_ for ImaR compared to parental cells supported our reasoning (Table 1 and Figure S8). Finally, it is worth noting that the IC_50_ of Doxorubicin for KU812 ImaR cells is 28 ± 5 nM, similar to the IC_50_ values obtained for AML cells (29 ± 12 nM for THP-1 and 18 ± 4 nM for HL-60).

## 3. Discussion

The here conducted multi-omics comparative analysis, in which we used proteomics and metabolomics profiling of a newly generated cell line model (KU812 ImaR) and its parental counterpart, unveiled the importance of the metabolic changes in the process of acquisition of TKI resistance and points towards putative metabolic vulnerabilities that can be exploited for the development of new chemotherapeutic strategies (summarized in Figure S9).

The initial metabolic phenotyping has been performed both in normoxia and hypoxia, to study changes due to TKI-resistance acquisition in a CML-specific microenvironment. Results showed that the main metabolic changes (i.e., increase in glycolysis and TCA cycle) identified in KU812 ImaR cells compared to parental cells are maintained in hypoxia. We have observed changes in complex I and IV activities under normoxia, which coincides with what is written in literature that hypoxia produces small adjustments by modifying individual proteins in complex I to IV [31].

In our study, we differentially analyzed the route taken by glucose towards various pathways. Our analyses support the fact that TKI-resistant cells became more glycolytic during the resistance development (Figure 3) and unveil the rewiring of glucose usage to other pathways different from glycolysis such as PPP, glycogen synthesis and SGOC metabolism. This aligns with published data showing that CML cells have a preference for glycolytic degradation of glucose to lactate [16,32], as well as with long-term imatinib resistant cells which increase glycolysis and PPP, using glycolysis to an even higher degree than parental cells [32]. It is worth noting that the extracellular acidification rate, a measurement of excreted H^+^, did not change between parental and TKI-resistant cells even though lactate production was increased in the TKI-resistant cells. We hypothesize that TKI-resistant cells enhance mechanisms to neutralize this pH decrease, for instance thorough the observed downregulation of CAs and upregulation of SLC4A7 transporter, or through lysosomes. Consistently, proteomic profiling highlighted the upregulation of LAMP1 and LAMP2, enzymes in charge of the integrity of lysosomes, and V-ATPase, which pumps protons across the lysosomal membrane using ATP to decrease lysosome pH [33] (Annex 1 in Supplementary Material). Therefore, our results and results from others, revealing the lysosome-mediated multidrug resistance to TKIs [34], propose that lysosome metabolism is enhanced in TKI-resistant cells, and further studies should be developed to better understand the role of lysosomes in TKI resistance phenotype.

Regarding the glucose rewiring, glycogen represents a major storage of high-energy glucose, which does not require ATP for activation. Glycogen degradation could be important for cell survival under stress conditions (i.e., drug treatment) [35], providing an easily accessible energy source. We have demonstrated that TKI-resistant cells exhibit higher glycogen storage capacity as well as a more active glycogen metabolism (Figure 3D,G). Moreover, the fact that the most strongly upregulated enzymes were PYGL and PGM1 suggests that glycogen metabolism is fine-tuned to ensure a higher glycogen storage coexisting with an enhanced PPP flux. In this sense, we further determined that the expression of the majority of PPP enzymes and activity of the two main enzymes (G6PD and TKT) were upregulated in the TKI-resistant cells (Figure 3D–F), leading us to conclude that KU812 ImaR cells have enhanced both oxidative and non-oxidative PPP, probably because this cell line may require more ribose-5-P, which plays a vital role for nucleotide synthesis, and NADPH which is essential for fatty acid synthesis, redox homeostasis and ROS scavenging [36]. These results agree with the enhanced PPP also observed by Noel et al. in K562 imatinib-resistant cells [32]. Furthermore, our results revealed that KU812 ImaR cells ensure an enhanced PPP flux thanks in part to the activation of glycogen synthesis and glycogenolysis, similarly to the results shown in a study performed with T-cells [37]. The inhibition of PYGL and G6PD did not substantially affect the cell viability of KU812 ImaR cells more, but it decreased the cell viability of both KU812 P and ImaR cells which open new avenues to be considered in drug repurposing for CML. 

Right now, the role of amino acid metabolism in TKI resistance has been poorly explored. Our results go in deep on the role that some amino acids metabolism may play in TKI resistance. Thus, we have observed that proteins involved in SGOC metabolism were upregulated (Figure 3D), as well as that TKI-resistant cells consumed more serine and methionine, and accumulated more glycine, than parental cells (Figure 3H–I). Thus, these data suggest that SGOC may be important for our TKI-resistant cells to accomplish DNA synthesis. This is also written in the literature, where recent studies have focused on the role of serine and glycine in protein synthesis, their contribution to anabolic pathways [38] and one-carbon metabolism [39]. Even though a more detrimental effect regarding the resistant cells was not observed, confirming the metabolic plasticity of KU812 ImaR cells, the low IC_50_ obtained for methotrexate and pemetrexed (Table 1) unveiled the SGOC pathway as an attractive therapy target to be further explored in new therapeutic strategies against CML. 

Glutamine is a well-known nutrient used by cancer cells to increase proliferation under metabolic stress conditions [40]. Our data showed that of all the metabolic pathways that glutamine can take, its contribution in proline synthesis is the most significant choice, suggesting its importance in resistance development in this cellular model (Figure 4C–F). Proline synthesis has been shown to fuel protein production for cell proliferation and to indirectly support biomass production by the generation of NADP^+^, inducing an increase in PPP activity [41]. Indeed, it is known that proline can be stored in collagen, the main component of extracellular matrix, and subsequently be re-mobilized during conditions of nutritional stress [25]. Interestingly, protein profiling of TKI-resistant cells exhibited an upregulation of collagen synthesis enzymes as compared to parental cells (Figure 4C). Thus, proline and collagen synthesis seem to be important for CML cells to develop TKI resistance. Likewise, our data also determined that glutathione, which is directly linked with glutamine metabolism, is required by TKI-resistant cells to both ROS scavenging (Figure 5) and detoxification of xenobiotics (i.e., carcinogens, anticancer drugs, metabolites, etc.) through S-glutathionylation [42]. Ezatiostat, a GSTP1 inhibitor, and RSL3, a GPX4 inhibitor which has been reported as a novel strategy to prevent tumor relapse [26], reduced cell viability of both parental and TKI-resistant cells (Table 1) with an IC_50_ in the low µM range unveiling glutathione metabolism as a putative target for the management of TKI resistance.

With regard to mitochondrial activity, in this study we observed a general upregulation of TCA cycle enzymes (Figure 6E) and an increased mitochondrial respiration (Figure 6A) in BCR-ABL1-independent TKI-resistant cells, although their activity of complex IV was less active (Figure 6H). However, only 36% of the main carbon sources (glucose/pyruvate, glutamine and fatty acids) were utilized for mitochondrial respiration by KU812 ImaR cells under normoxia and 54% under hypoxia (Figure 6D). Furthermore, our results prove that one of these alternative contributors to mitochondrial respiration is GP via the GP shuttle (Figure 8A). This GP shuttle, captained by GPD2 enzyme, is also contributing to consume oxygen and synthesize ATP, thus avoiding the activity of complex I and II but also contributing to the higher mitochondrial respiration observed in KU812 ImaR cells. Our results contrast with those published in imatinib resistant CML cells due to BCR-ABL1 mutations, where a reduced activity of the ETC complexes was described [22]. 

It is also worth mentioning that KU812 ImaR cells were 12-fold more vulnerable to doxorubicin, a standard treatment for AML patients. However, the mechanisms behind this differential effect of doxorubicin are not well understood and further investigations are required to better comprehend why the here studied BCR-ABL1-independent TKI-resistant cells were more sensitive to doxorubicin than KU812 Parental cells. In any case, the use of doxorubicin also appears as a new putative opportunity to treat CML TKI-resistant patients.

Altogether, the obtained results from targeting selected key players in carbon-metabolism pathways enhanced in KU812 ImaR cells using single-hit strategies, reveal that the metabolic plasticity of the KU812 ImaR cells is so high that punctual inhibitions in key enzymes are not enough to compromise their cell viability. Results also show that both KU812 P and ImaR cells were highly sensitive to the inhibition of one-carbon metabolism and glutathione metabolism (IC_50_ values in the range of nM) making methotrexate, pemetrexed and RSL-3, as drug candidates to be considered in future studies for the management of TKI-resistance. Further investigations in other CML cell lines and primary patient-derived materials could thus reveal new approaches for the treatment of TKI-resistant CML. 

## 4. Materials and Methods

### 4.1. Chemicals and Reagents

Mitochondrial GPDH Inhibitor was from Calbiochem, shin2 from Glixx laboratories, and CP-320626 was kindly provided by Dr. Loranne Agius, Newcastle, UK. Etomoxir was from Cayman Chemical, antibiotic (10,000 U/mL penicillin, 10 mg/mL streptomycin), PBS and Trypsin EDTA solution C (0.05% trypsin–0.02% EDTA) were from Biological Industries (Kibbutz Beit Haemet, Israel), and fetal bovine serum was from Invitrogen (Carlsbad, CA, USA). The rest of reagents were from Sigma-Aldrich (St. Louis, MO, USA). 

### 4.2. Cell Culture and Development of Imatinib Resistant Cell Line

KU812 cell line was obtained through the American Type Culture Collection and was grown in RPMI-1640 media supplemented with 10% fetal calf serum, 4 mM glutamine, and 1% penicillin/streptomycin at 37 °C and 5% CO_2_. For hypoxia, cells were kept in a hypoxia chamber (XVivo, Biospherix, Parish, NY, USA) containing 1% oxygen and 5% CO_2_ for at least 5 days prior to performance of experiments. For KU812 imatinib treated cells, KU812 parental cells were treated with 0.08 µM of imatinib for 72 h prior to performance of experiments.

To generate imatinib resistant cells, KU812 parental cells were treated with increasing concentrations of imatinib (0.074–1 µM), using an increase of 0.017 µM every week depending on cell health. Cells were considered resistant to imatinib when their doubling time in the presence of 1 µM imatinib was almost the same as that of the parental cells in the absence of imatinib. Acquisition of resistance was verified by determining the IC_50_ concentration of imatinib after 72 h of treatment. Imatinib resistant cells were maintained with additional supplementation of 0.2 µM imatinib. 

### 4.3. Cell Viability Assays

IC_50_ concentration was determined from dose-response curves where cells were incubated with increasing concentrations of a drug, and determining their cell viability using Cell Titer-Glo^®^ Luminescent Cell Viability Assay (Promega, Madison, WI, USA) or Hoechst (HO33342) staining, both according to manufacturer’s instructions. 

### 4.4. Measurement of Enzyme Activities, Consumption and Production Rates of Metabolites and Amino Acids and Polyamines Intracellular Concentration

Specific enzyme activities of G6PD, HK, lactate dehydrogenase (LDH), PK and TKT were also determined by using NAD(P)H-coupled enzymatic reactions [43].

Consumption and production rates of metabolites were determined measuring metabolite concentration in incubation media at the beginning and at the end of incubation time, and correcting the absolute consumption by time and cell number assuming exponential cell growth. 

Amino acids and polyamines intracellular concentrations were determined from cell lysates using EtOH:PBS 85:15 and correcting concentrations by cell lysate protein content. 

Glucose, lactate, glutamate and glutamine concentrations were measured using NAD(P)H-coupled enzymatic assays in a COBAS Mira Plus spectrophotometer (Horiba ABX, Kyoto, Japan) [43]. 

The concentration of amino acids and polyamines in incubation media and cell lysates was determined using the AbsoluteIDQ™ p180 Kit (Biocrates Life Sciences, Innsbruck, Austria) and the AB Sciex 4000 QTRAP MS/MS mass spectrometer coupled to an Agilent HUPLC 1200, according to the manufacturer’s instructions. Analyst and the MetIDQ™ software packages were used to analyze the obtained data and calculate metabolite concentrations. 

### 4.5. Measurement of Glycogen, ROS and Glutathione Intracellular Levels

Measurement of the glycogen content was carried out by gas chromatography/mass spectrometry (GC/MS) using [U-^13^C-D_7_]-glucose as recovery standard and internal standard quantification procedures, as described in [44]. Glucose from glycogen was corrected by cell number.

Total intracellular ROS levels were determined after 5 min of incubation of cells with 5 µM 2′-7′-dichlorodihydrofluorescein diacetate (DCFDA, Invitrogen) in PBS supplemented with 4 mM glutamine and 10 mM glucose, and reading the emitted fluorescence by flow cytometry [45]. 

Total glutathione content was determined using the glutathione reductase enzymatic method [45]. 

### 4.6. Nuclear Magnetic Resonance (NMR) Analysis

30 million KU812 parental and ImaR cells were incubated for 24 h in glucose/glutamine-free media supplemented with 11 mM [U-^13^C]-glucose + 2 mM glutamine for the glucose tracer condition or 2 mM [U-^13^C]-glutamine + 11 mM glucose for the glutamine tracer condition. At the end of incubation, cells were treated as described in [46], and 1D and 2D NMR were used to measure and quantify individual compounds [46,47]. Their assignment was added by the isotope enrichment. Finally, metabolites identification was performed using Chenomx software and metabolite quantification and label incorporation analysis was performed in ‘NMRLab’ [48].

### 4.7. Western Blotting

Protein cell lysates were done using RIPA buffer and Western blots were performed using standard procedures [45]. Antibodies against ALDH18A1 (P5CS), ALDH4A1 (P5CDH), HK I, HK II, HKIII, P5CRL (PYCR) and GL Syn (all 1:1000 dilution) and Lamin-B (1:10,000) were obtained from Santa Cruz Biotechnology; PRODH and GLS2 (both 1:1000) were from GeneTex; TATABOX and GLS1 (both 1:1000) were from Abcam; and TKT (1:1000) was from Sigma. Lamin-B and TATABOX were used interchangeably as loading controls.

### 4.8. Oxygen Consumption Rate (OCR) and Extracellular Acidification Rate (ECAR)

OCR and ECAR of intact KU812 Parental and ImaR cells were determined using a XF96 Extracellular Flux Analyzer (Agilent, Santa Clara, CA, USA). For that, a total of 2 × 10^5^ cells in 200 µL of media or buffer were plated in each well of Seahorse 96-well cell culture plates and equilibrated for 30 min before analysis. For Mitostress test, cells were seeded in Dulbecco’s Modified Eagle’s Medium (DMEM D5030) supplemented with 10 mM glucose and 4 mM glutamine, and sequential injections of: 2.5 µM oligomycin; 0.3 µM carbonyl cyanide m-chlorophenylhydrazone (CCCP); 0.3 µM of CCCP + 2 mM of Pyruvate; and 1 µg/mL antimycin A + 1 µM rotenone, were added. For glycolysis test, cells were seeded in Krebs Henseleit buffer (111 mM NaCl, 4.7 mM KCl, 1.25 mM CaCl2, 2 mM MgSO_4_, 1.2 mM Na_2_HPO_4_) supplemented with 10 mM glucose, and sequential injections of: 5 mM glucose; 5 mM glucose (10 mM in total); 2.5 µM oligomycin; and 100 mM 2-deoxyglucose (2-DG), were added. To quantify total fatty acid, glutamine and glucose/pyruvate contribution to mitochondrial respiration, cells were seeded in DMEM D5030 supplemented with 10 mM glucose and 4 mM glutamine, and sequential injections of: 2 mM UK5099 (to inhibit the mitochondrial pyruvate transporter); 4 mM etomoxir (to block carnitine palmitoyl transferase 1A); 3 mM BPTES (to antagonize glutaminase); and 2.5 µM oligomycin, were added.

### 4.9. Oximetry for KU812 Parental and Resistant Cell Lines

Oxygen flow in KU812 Parental, treated, and resistant cells was measured using a two-channel, high-resolution Oxygraph respirometer (Oroboros, Innsbruck, Austria), as described in [49]. Briefly, two million cells were collected and washed once in phosphate buffered saline (PBS), centrifuged, and finally resuspended in MiR05 (3 mM MgCl_2_, 0.5 mM EGTA, 20 mM taurine, 10 mM KH_2_PO_4_, 60 mM K-lactobionate, 110 mM sucrose, 20 mM HEPES, and 1 g/L bovine serum albumin) for permeabilized-cell approaches and in RPMI-1640 for intact-cell approach. For intact cell analysis, routine respiration was recorded and then 2.5 µM oligomycin were added to inhibit the ATP synthase, which allows one to measure leak respiration. The electron transfer system (ETS) capacity was evaluated by titration with CCCP uncoupler in 0.5 µM steps until a maximum flow was reached. Respiration was inhibited by 0.5 µM rotenone and 2.5 µM antimycin A to determine residual oxygen consumption (ROX). For digitonin-permeabilized cells, oxygen flow was measured before digitonin treatment in respiration medium MiR05 (Oroboros Instruments GmbH, Innsbruck, Austria), and after successive addition of substrates and inhibitors as follows: (i) To measure complex I activity: 10 mM Glutamate, 2 mM Malate, 5 mM ADP, and 5 mM pyruvate; (ii) To measure complex II activity: 10 mM glycerol-3-phosphate, 5 mM ADP and 10 mM succinate; (iii) Fatty acid approach: 0.04 mM palmitoyl-CoA carnitine plus 0.1 mM malate, 5 mM ADP and 1.9 mM malate. All respiratory coupling states were corrected for ROX, which was obtained after the addition of 2.5 µM antimycin A. Finally, 5 mM ascorbate plus 0.5 mM N,N,N′,N′-tetramethyl-p-phenylendiamine dihydrochloride (TMPD) were used as substrates to assess complex IV activity. Mitochondrial membrane integrity was verified after the addition of 10 µM cytochrome c, and changes were always lower than 10%. Data were analyzed using DatLab7.4.0.4 software (Oroboros, Innsbruck, Austria). Oxygen flow is expressed as picomoles per second per million cells.

### 4.10. SILAC-Mediated Proteomic Data Analysis

SILAC-labelling was performed using SILAC RPMI (Thermo Fisher, Madison, WI, USA, #88365) supplemented with 10% dialyzed FBS, 100 U/mL Penicillin, 100 µg/mL Streptomycin and the respective SILAC amino acids (all from Cambridge Isotopes, Tewksbury, MA, USA). “Light” SILAC medium contained [^12^C_6_^14^N_4_]-L-arginine and [^12^C_6_^14^N_2_]-L-lysine and “heavy” SILAC medium contained [^13^C_6_^15^N_4_]-L-arginine and [^13^C_6_^15^N_2_]-L-lysine. For KU812 Parental and ImaR cell lysis, cells were washed with cold PBS and lysed in 0.5% Nonidet P-40 buffer containing 50 mM Tris/HCl, pH 7.5, 150 mM NaCl, 1 mM Na_3_VO_4_, 5 mM NaF, and the Complete™, Mini, EDTA-free Protease Inhibitor Cocktail (Sigma-Aldrich, St. Louis, MO, USA). For protein expression profiling, lysates of cells differentially labeled during SILAC were mixed in equimolar ratios and separated by SDS-PAGE using precast Bis-Tris minigels (NuPAGE Novex 4–12%, Life Technologies, Carlsbad, CA, USA). After protein visualization by staining with Coomassie Brilliant Blue (SERVA, Heidelberg, Germany), each gel lane was cut into 23 slices. The separated proteins were reduced with DTT (Sigma-Aldrich) and alkylated with iodoacetamide (Sigma-Aldrich). After overnight in-gel protein digestion with trypsin (Serva), peptides were extracted from the gel matrix and analyzed by liquid chromatography/mass spectrometry (LC/MS). A Q Exactive mass spectrometer was operated in a data-dependent acquisition mode selecting the top 12 most abundant precursor ions for higher energy collisional dissociation (HCD) with an isolation width of 2 m/z and an NCE setting of 28%. Survey spectra from *m*/*z* 350–1600 were acquired with an MS resolution setting of 70,000 FWHM at *m*/*z* 200 and product ion spectra with a MS/MS resolution of 17,500 in the Orbitrap analyzer. AGC target values and maximum injection times for MS and MS/MS were set to 1 × 10^6^ in 60ms and 2 × 10^5^ in 60 ms, respectively. Raw data files from LC-MS/MS measurements were processed using the MaxQuant software (version 1.6.0.1, MPI for Biochemistry) [50]. MS/MS spectra were searched against the UniProtKB/Swiss-Prot human database containing 88,993 protein entries (downloaded November 2016) supplemented with 245 frequently observed contaminants with the Andromeda search engine [51]. Precursor and fragment ion mass tolerances were set to 6 and 20 ppm after initial recalibration, respectively. Protein N-terminal acetylation and methionine oxidation were allowed as variable modifications. Cysteine carbamidomethylation was defined as a fixed modification. Minimal peptide length was set to seven amino acids, with a maximum of two missed cleavages. The false discovery rate (FDR) was set to 1% on both the peptide and the protein level using a forward-and-reverse concatenated decoy database approach. For SILAC quantitation, multiplicity was set to two for double labelling (Lys+0/Arg+0, Lys+8/Arg+10) and at least two ratio counts were required for peptide quantitation. Both the “match between runs” and “re-quantify” options of MaxQuant were enabled.

Subsequent evaluation of MaxQuant output data was conducted with the Perseus software (version 1.6.0.7, MPI for Biochemistry) [52]. After removal of potential false-positive entries and potential contaminants, the SILAC ratios were logarithmized and filtered for valid values in each measurement. To assign regulated proteins, ratio thresholds were determined by subjecting the SILAC ratios of the whole population of identified proteins to an outlier analysis based on an FDR < 5% and Benjamini-Hochberg correction for multiple hypothesis testing [50]. Next, Fold change (FC) values were calculated using FC threshold of 1.5. Finally, log_2_(FC) values were integrated and visualized in specific pathways using Pathview Web [53] and specific biological processes using PANTHER [54].

## 5. Conclusions

In conclusion, the comprehensive multi-omics approach used here revealed that CML cells that acquire TKIs resistance developed a strong metabolic rewiring. We demonstrated that this acquired resistance could be counteracted using drugs that target the identified metabolic key players. Nevertheless, we could also show that high metabolic plasticity contradicts the efficacy of a single-hit strategy, so that treatment of TKI-resistant CML requires a combination of inhibitors against vulnerable metabolic pathways.

## Figures and Tables

**Table 1:** The half-maximal inhibitory concentration (IC_50_) values of KU812 Parental and ImaR cells upon inhibitor treatments.

Inhibitor	Target	Pathway	KU812 Parental IC_50_ (µM)	KU812 ImaR IC_50_ (µM)	DRI
RSL-3	GPX4	Glutathione metabolism	0.64 ± 0.09	0.58 ± 0.10	1.1
CP-320626	PYG	Glycogenolysis	33 ± 17	32 ± 14	1.0
CP-91149	PYG	Glycogenolysis	46 ± 15	76 ± 7	0.6
DAB	PYG	Glycogenolysis	868 ± 218	588 ± 116	1.4
Methotrexate	DHFR	One-carbon metabolism	0.012 ± 0.03	0.017 ± 0.005	0.7
Pemetrexed	TYMS, DHFR and GARFT	One-carbon metabolism	0.042 ± 0.014	0.079 ± 0.015	0.5
Shin2	SHMT	One-carbon metabolism	33.8 ± 4.6	40.3 ± 2.1	0.8
DHEA	G6PD	PPP	11.6 ± 1.2	8.1 ± 1.3	1.4
Ezatiostat	GSTP1	S-glutathionylation	14.5 ± 5.8	7.2 ± 0.2	2.0
Doxorubicin	Topoisomerase II, mitochondrial respiration and OXPHOS	Multitarget and AML standard treatment	0.36 ± 0.09	0.028 ± 0.005	12

The half-maximal inhibitory concentration (IC_50_) and Drug resistance index (DRI) are shown. Mean ± SD for *n* = 3. Abbreviations: DAB, 1,4-dideoxy-1,4-imino-d-arabinitol; DHEA, deshidroepiandrosterona; DHFR, dihydrofolate reductase; DRI, drug resistant index; G6PD, glucose-6-phosphate dehydrogenase; GARFT, glycinamide ribonucleotide; GSTP1, glutathione S-transferase P1; GPX4, glutathione peroxidase 4; ImaR, imatinib resistant; PPP, pentose phosphate pathway; PYG, glycogen phosphorylase; SHMT, serine hydroxymethyltransferase; and TYMS, thymidylate synthetase.

## Supplementary Materials

The following are available online at https://www.mdpi.com/2072-6694/12/11/3443/s1, Figure S1: BCR-ABL1 mutation analysis, Figure S2: Cell cycle distribution of KU812 Parental and KU812 ImaR cells determined by flow cytometry, Figure S3: Glycolytic profile, protein profile differences of protein associated with H^+^ balance and Crabtree effect of KU812 Imatinib resistant compared to KU812 Parental cells, Figure S4: ^13^C Glucose label incorporation in KU812 Parental and ImaR cells under normoxia measured by NMR, Figure S5: ^13^C Glutamine label incorporation in KU812 Parental and ImaR cells under normoxia measured by NMR, Figure S6: Activity of the different electron transport chain complexes of KU812 Imatinib-treated vs. KU812 Parental cells, Figure S7: Diagram of the oxidative phosphorylation and glycerol-3-Phosphate shuttle, Figure S8: Effects of different metabolic inhibitors and AML conventional drugs in KU812 Parental and ImaR cell viability in normoxia, Figure S9: Schematic diagram of the metabolic reprogramming upon TKI resistance acquisition, Table S1: Exchange flux values of KU812 Parental and ImaR in normoxia after specific incubation times, Table S2: Exchange flux values of KU812 Parental and ImaR in hypoxia (1% O_2_) after specific incubation times, Table S3: Intracellular content of KU812 Parental and ImaR in normoxia after 24h incubation time, Table S4: Intracellular content of KU812 Parental and ImaR in hypoxia (1% O_2_) after 24h incubation time, Annex 1: Supplementary_MS_SILAC_file.

## References

[1] Sawyers C.L. (1999). Chronic myeloid leukemia. N. Engl. J. Med..

[2] Rowley J.D. (1973). A New Consistent Chromosomal Abnormality in Chronic Myelogenous Leukaemia identified by Quinacrine fluorescence and Giemsa staining. Nature.

[3] Druker B.J., Talpaz M., Resta D.J., Peng B., Buchdunger E., Ford J.M., Lydon N.B., Kantarjian H., Capdeville R., Ohno-Jones S. (2001). Efficacy and Safety of a Specific Inhibitor of the Bcr-Abl Tyrosine. N. Engl. J. Med..

[4] Chan W.Y., Lau P.M., Yeung K.W., Kong S.K. (2018). The second generation tyrosine kinase inhibitor dasatinib induced eryptosis in human erythrocytes—An in vitro study. Toxicol. Lett..

[5] Breccia M., Alimena G. (2010). Nilotinib: A second-generation tyrosine kinase inhibitor for chronic myeloid leukemia. Leuk. Res..

[6] O’Brien S.G., Guilhot F., Larson R.A., Gathmann I., Baccarani M., Cervantes F., Cornelissen J.J., Fischer T., Hochhaus A., Hughes T. (2003). Imatinib Compared with Interferon and Low-Dose Cytarabine for Newly Diagnosed Chronic-Phase Chronic Myeloid Leukemia. N. Engl. J. Med..

[7] Branford S., Hughes T. (2006). Detection of BCR-ABL Mutations and Resistance to Imatinib Mesylate. Methods Mol. Med..

[8] Jabbour E., Kantarjian H., Jones D., Talpaz M., Bekele N., O’Brien S., Zhou X., Luthra R., Garcia-Manero G., Giles F. (2006). Frequency and clinical significance of BCR-ABL mutations in patients with chronic myeloid leukemia treated with imatinib mesylate. Leukemia.

[9] Gorre M.E., Mohammed M., Ellwood K., Hsu N., Paquette R., Rao P.N., Sawyers C.L. (2001). Clinical resistance to STI-571 cancer therapy caused by BCR-ABL gene mutation or amplification. Science.

[10] Illmer T., Schaich M., Platzbecker U., Freiberg-Richter J., Oelschlägel U., von Bonin M., Pursche S., Bergemann T., Ehninger G., Schleyer E. (2004). P-glycoprotein-mediated drug efflux is a resistance mechanism of chronic myelogenous leukemia cells to treatment with imatinib mesylate. Leukemia.

[11] Vander Heiden M.G., Cantley L.C., Thompson C.B. (2009). Understanding the Warburg Effect: Cell Proliferation. Science.

[12] Zheng J. (2012). Energy metabolism of cancer: Glycolysis versus oxidative phosphorylation (Review). Oncol. Lett..

[13] Gouirand V., Guillaumond F., Vasseur S. (2018). Influence of the Tumor Microenvironment on Cancer Cells Metabolic Reprogramming. Front. Oncol..

[14] Zaal E.A., Berkers C.R. (2018). The Influence of Metabolism on Drug Response in Cancer. Front. Oncol..

[15] Boren J., Cascante M., Marin S., Comín-Anduix B., Centelles J.J., Lim S., Bassilian S., Ahmed S., Lee W.N., Boros L.G. (2001). Gleevec (STI571) Influences Metabolic Enzyme Activities and Glucose Carbon Flow toward Nucleic Acid and Fatty Acid Synthesis in Myeloid Tumor Cells. J. Biol. Chem..

[16] Gottschalk S., Anderson N., Hainz C., Eckhardt S.G., Serkova N.J. (2004). Imatinib (STI571)-mediated changes in glucose metabolism in human leukemia BCR-ABL-positive cells. Clin. Cancer Res..

[17] Breccia M., Alimena G. (2009). The metabolic consequences of imatinib mesylate: Changes on glucose, lypidic and bone metabolism. Leuk. Res..

[18] Poliaková M., Aebersold D.M., Zimmer Y., Medová M. (2018). The relevance of tyrosine kinase inhibitors for global metabolic pathways in cancer. Mol. Cancer.

[19] Maharsy W., Aries A., Mansour O., Komati H., Nemer M. (2014). Ageing is a risk factor in imatinib mesylate cardiotoxicity. Eur. J. Heart Fail..

[20] Bouitbir J., Panajatovic M.V., Frechard T., Roos N.J., Krähenbühl S. (2020). Imatinib and Dasatinib Provoke Mitochondrial Dysfunction Leading to Oxidative Stress in C2C12 Myotubes and Human RD Cells. Front. Pharmacol..

[21] Zhao F., Mancuso A., Bui T.V., Tong X., Gruber J.J., Swider C.R., Sanchez P.V., Lum J.J., Sayed N., Melo J.V. (2010). Imatinib resistance associated with BCR-ABL upregulation is dependent on HIF-1alpha-induced metabolic reprograming. Oncogene.

[22] Kluza J., Jendoubi M., Ballot C., Dammak A., Jonneaux A., Idziorek T., Joha S., Dauphin V., Malet-Martino M., Balayssac S. (2011). Exploiting mitochondrial dysfunction for effective elimination of imatinib-resistant leukemic cells. PLoS ONE.

[23] Favaro E., Bensaad K., Chong M.G., Tennant D.A., Ferguson D.J.P., Snell C., Steers G., Turley H., Li J.-L., Günther U.L. (2012). Glucose utilization via glycogen phosphorylase sustains proliferation and prevents premature senescence in cancer cells. Cell Metab..

[24] Hussien R., Brooks G.A. (2011). Mitochondrial and plasma membrane lactate transporter and lactate dehydrogenase isoform expression in breast cancer cell lines. Physiol. Genom..

[25] Phang J.M., Liu W., Hancock C.N., Fischer J.W. (2015). Proline metabolism and cancer: Emerging links to glutamine and collagen. Curr. Opin. Clin. Nutr. Metab. Care.

[26] Hangauer M.J., Viswanathan V.S., Ryan M.J., Bole D., Eaton J.K., Matov A., Galeas J., Dhruv H.D., Berens M.E., Schreiber S.L. (2017). Drug-tolerant persister cancer cells are vulnerable to GPX4 inhibition. Nature.

[27] Weich N., Ferri C., Moiraghi B., Bengió R., Giere I., Pavlovsky C., Larripa I.B., Fundia A.F. (2016). GSTM1 and GSTP1, but not GSTT1 genetic polymorphisms are associated with chronic myeloid leukemia risk and treatment response. Cancer Epidemiol..

[28] Mráček T., Drahota Z., Houštěk J. (2013). The function and the role of the mitochondrial glycerol-3-phosphate dehydrogenase in mammalian tissues. Biochim. Biophys. Acta Bioenerg..

[29] Tacar O., Sriamornsak P., Dass C.R. (2013). Doxorubicin: An update on anticancer molecular action, toxicity and novel drug delivery systems. J. Pharm. Pharmacol..

[30] Wallace K.B., Sardão V.A., Oliveira P.J. (2020). Mitochondrial Determinants of Doxorubicin-Induced Cardiomyopathy. Circ. Res..

[31] Fuhrmann D.C., Brüne B. (2017). Mitochondrial composition and function under the control of hypoxia. Redox Biol..

[32] Noel B.M., Ouellette S.B., Marholz L., Dickey D., Navis C., Yang T.-Y., Nguyen V., Parker S.J., Bernlohr D., Sachs Z. (2019). Multi-omic profiling of TKI resistant K562 cells suggests metabolic reprogramming to promote cell survival. J. Proteome Res..

[33] Saftig P., Klumperman J. (2009). Lysosome biogenesis and lysosomal membrane proteins: Trafficking meets function. Nat. Rev. Mol. Cell Biol..

[34] De Klerk D.J., Honeywell R.J., Jansen G., Peters G.J. (2018). Transporter and lysosomal mediated (Multi)drug resistance to tyrosine kinase inhibitors and potential strategies to overcome resistance. Cancers.

[35] Zois C.E., Harris A.L. (2016). Glycogen metabolism has a key role in the cancer microenvironment and provides new targets for cancer therapy. J. Mol. Med..

[36] Patra K.C., Hay N. (2014). The pentose phosphate pathway and cancer. Trends Biochem. Sci..

[37] Olivas J., Horng T. (2018). Sugar fuels T-cell memory. Nat. Cell Biol..

[38] Locasale J.W. (2013). Serine, glycine and the one-carbon cycle: Cancer metabolism in full circle. Nat. Rev. Cancer.

[39] Mehrmohamadi M., Liu X., Shestov A.A., Locasale J.W. (2014). Characterization of the Usage of the Serine Metabolic Network in Human Cancer. Cell Rep..

[40] Choi Y.-K., Park K.-G. (2018). Targeting glutamine metabolism for cancer treatment. Biomol. Ther..

[41] Tanner J.J., Fendt S.-M., Becker D.F. (2018). The Proline Cycle as a Potential Cancer Therapy Target. Biochemistry.

[42] Armstrong R.N., Morgenstern R., Board P.G., McQueen C.A. (2018). Glutathione Transferases. Comprehensive Toxicology.

[43] De Atauri P., Benito A., Vizán P., Zanuy M., Mangues R., Marín S., Cascante M. (2011). Carbon metabolism and the sign of control coefficients in metabolic adaptations underlying K-ras transformation. Biochim. Biophys. Acta Bioenerg..

[44] Vizán P., Sánchez-Tena S., Alcarraz-Vizán G., Soler M., Messeguer R., Pujol M.D., Paul Lee W.N., Cascante M. (2009). Characterization of the metabolic changes underlying growth factor angiogenic activation: Identification of new potential therapeutic targets. Carcinogenesis.

[45] Aguilar E., Marin de Mas I., Zodda E., Marín S., Morrish F., Selivanov V., Meca-Cortés Ó., Delowar H., Pons M., Izquierdo I. (2016). Metabolic reprogramming and dependencies associated with epithelial cancer stem cells uncoupled from epithelial-mesenchymal transition. Stem Cells.

[46] Carrigan J.B., Reed M.A.C., Ludwig C., Khanim F.L., Bunce C.M., Günther U.L. (2016). Tracer-Based Metabolic NMR-Based Flux Analysis in a Leukaemia Cell Line. ChemPlusChem.

[47] Alshamleh I., Krause N., Richter C., Kurrle N., Serve H., Günther U.L., Schwalbe H. (2020). Real-Time NMR Spectroscopy for Studying Metabolism. Angew. Chem. Int. Ed. Engl..

[48] Gunther U.L., Ludwig C., Rüterjans H. (2000). NMRLAB-Advanced NMR data processing in Matlab. J. Magn. Reson..

[49] Doerrier C., Garcia-Souza L.F., Krumschnabel G., Wohlfarter Y., Meszaros A.T., Gnaiger E. (2018). High-Resolution FluoRespirometry and OXPHOS Protocols for Human Cells, Permeabilized Fibers from Small Biopsies of Muscle, and Isolated Mitochondria. Methods Mol. Biol..

[50] Cox J., Mann M. (2008). MaxQuant enables high peptide identification rates, individualized p.p.b.-range mass accuracies and proteome-wide protein quantification. Nat. Biotechnol..

[51] Cox J., Neuhauser N., Michalski A., Scheltema R.A., Olsen J.V., Mann M. (2011). Andromeda: A peptide search engine integrated into the MaxQuant environment. J. Proteome Res..

[52] Tyanova S., Temu T., Sinitcyn P., Carlson A., Hein M.Y., Geiger T., Mann M., Cox J. (2016). The Perseus computational platform for comprehensive analysis of (prote)omics data. Nat. Methods.

[53] Luo W., Pant G., Bhavnasi Y.K., Blanchard S.G., Brouwer C. (2017). Pathview Web: User friendly pathway visualization and data integration. Nucleic Acids Res..

[54] Mi H., Muruganujan A., Huang X., Ebert D., Mills C., Guo X., Thomas P.D. (2019). Protocol Update for large-scale genome and gene function analysis with the PANTHER classification system (v.14.0). Nat. Protoc..

