# Supplementary material for: Metabolic Plasticity Is an Essential Requirement of Acquired Tyrosine Kinase Inhibitor Resistance in Chronic Myeloid Leukemia
Source: Cancers (Basel). 2020 Nov 19;12(11):3443. doi: 10.3390/cancers12113443 (PMC7699488; doi:10.3390/cancers12113443)

Supplementary Materials:

Metabolic Plasticity Is An Essential Requirement of Acquired Tyrosine Kinase Inhibitor Resistance in Chronic Myeloid Leukemia

Miriam G. Contreras Mostazo, Nina Kurrle, Marta Casado, Dominik Fuhrmann, Islam Alshamleh, Björn Háupl, Paloma Martín-Sanz, Bernhard Brüne, Hubert Serve, Harald Schwalbe, Frank Schnuetgen, Silvia Marin and Marta Cascante

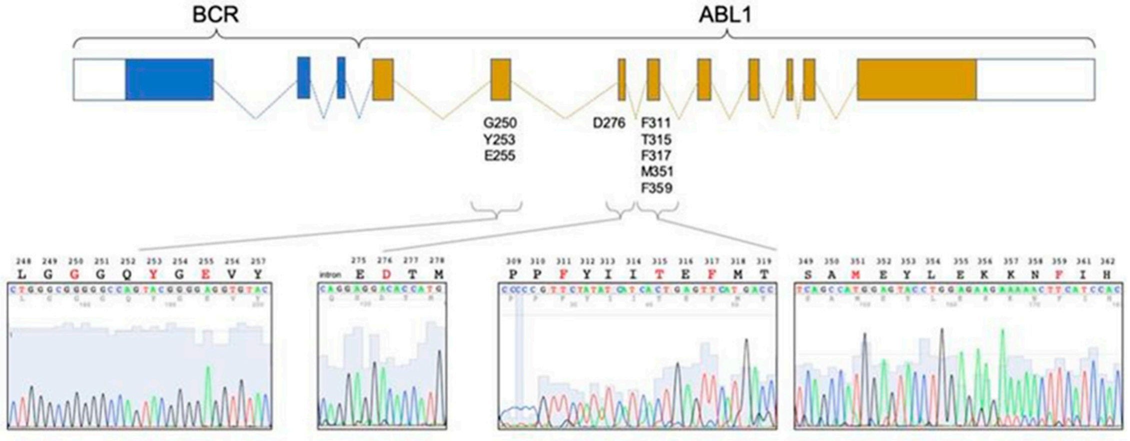

Figure S1. BCR-ABL1 mutation analysis. The nine indicated mutations account for 60% to 70% of all imatinib-resistant mutations. Exon 4,5 and 6 of ABL1 were amplified as explained in Materials and Methods and Sanger sequenced.

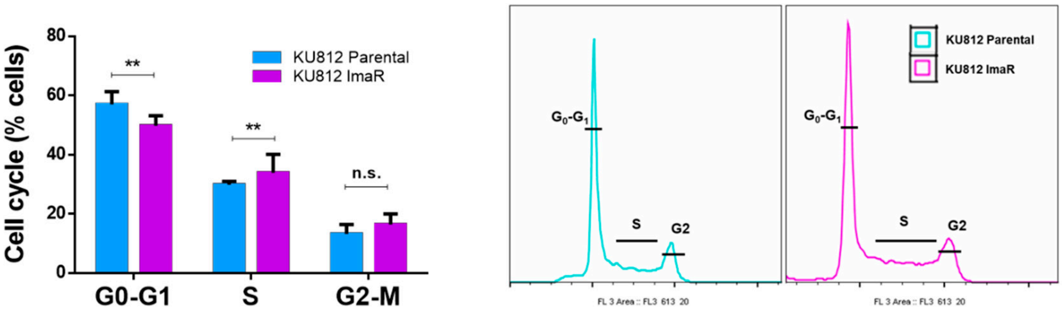

Figure S2. Cell cycle distribution of KU812 Parental and KU812 ImaR cells determined by flow cytometry. Figure shows one representative experiment (mean ± SD for n = 3 of n = 2 independent experiments). In addition, significance was determined by two-tailed independent sample Welch's t-tests. Statistically significant differences between KU812 Parental and ImaR cells were indicated as p < 0.01 (**) and n.s. = non-significant differences.

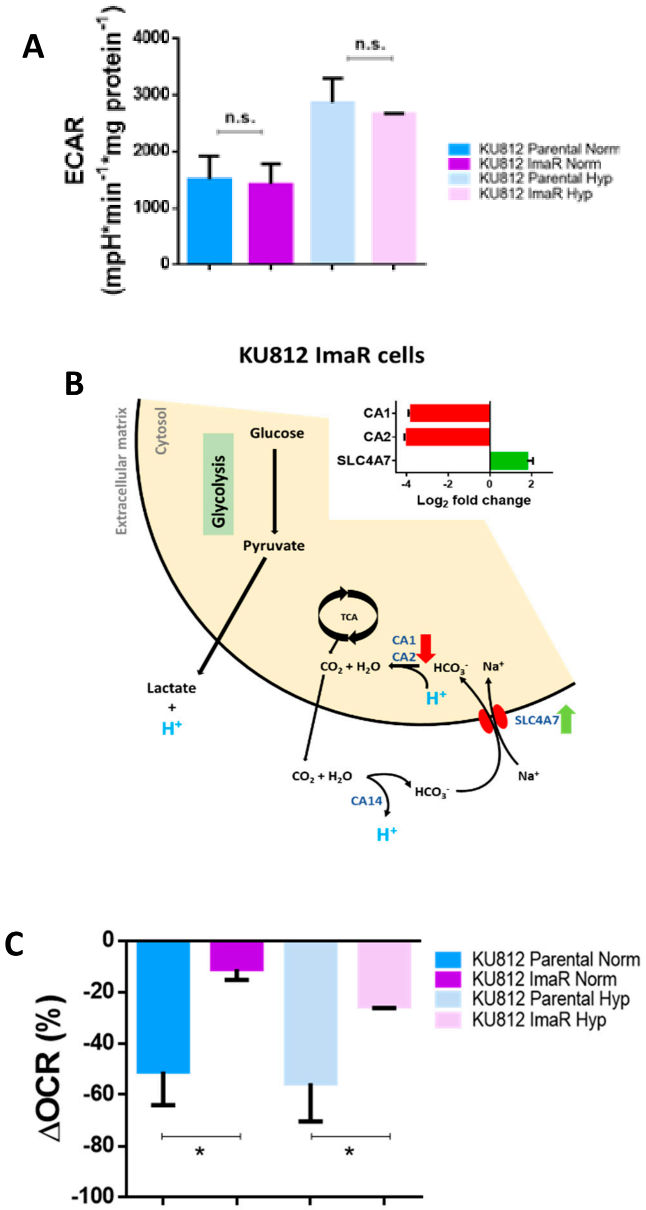

Figure S3. Glycolytic profile, protein profile differences of protein associated with H+ balance and Crabtree effect of KU812 Imatinib resistant compared to KU812 Parental cells. A) ECAR after glucose addition calculated using ECAR values from Figure 3C. B) The protein expression profiling of the carbonic anhydrase (CA) 1 and 2, and the sodium bicarbonate cotransporter (SLC4A7) obtained under normoxia using SILAC proteomic experiments. Log2 fold change values were calculated and represented by green colour = protein upregulation; and red = protein downregulation. Data are provided as mean ± SD of n=2 of one representative experiment C) Crabtree effect determined by adding 10 mM glucose to glucose-deprived media. Values of ΔOCR are expressed as % of the basal OCR values in absence of glucose. Data are provided as mean ± SD of n=3. Significance was determined by two-tailed independent sample Student's t-test. Statistical differences between KU812 P vs. ImaR cells under normoxic or hypoxic incubation conditions are indicated as $p<0.05$ (*) and $p\geq0.05$ (n.s.=non-significant differences).

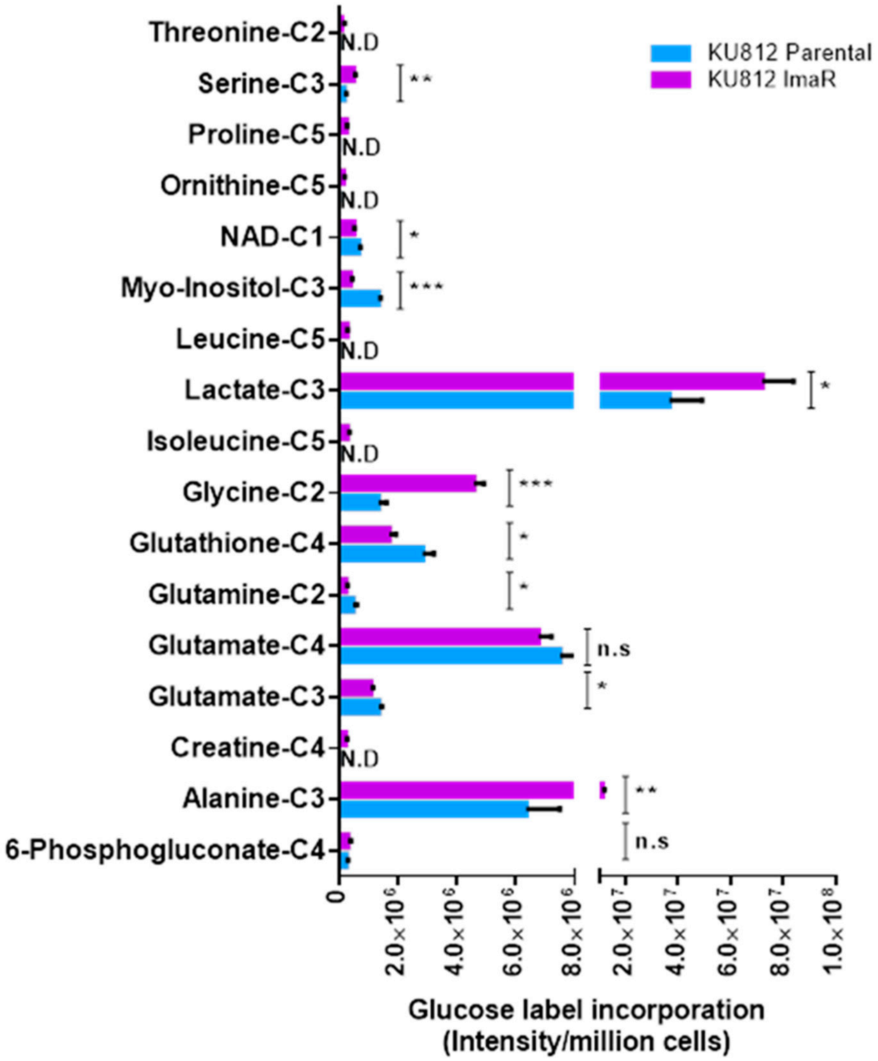

Figure S4. ^{13}C Glucose label incorporation in KU812 Parental and ImaR cells under normoxia measured by NMR. Intensity values were normalized by million cells. Figure shows one representative experiment (mean ± SD for *n* = 3 replicates). Significance was determined by two-tailed independent sample Student's t-tests. Statistically significant differences between KU812 Parental and KU812 ImaR cells were indicated as $p < 0.05$ (*), $p < 0.01$ (**), $p < 0.001$ (***), and $p \geq 0.05$ (n.s. = non-significant differences). Abbreviations: ImaR, imatinib resistant; N.D., non-detected metabolites.

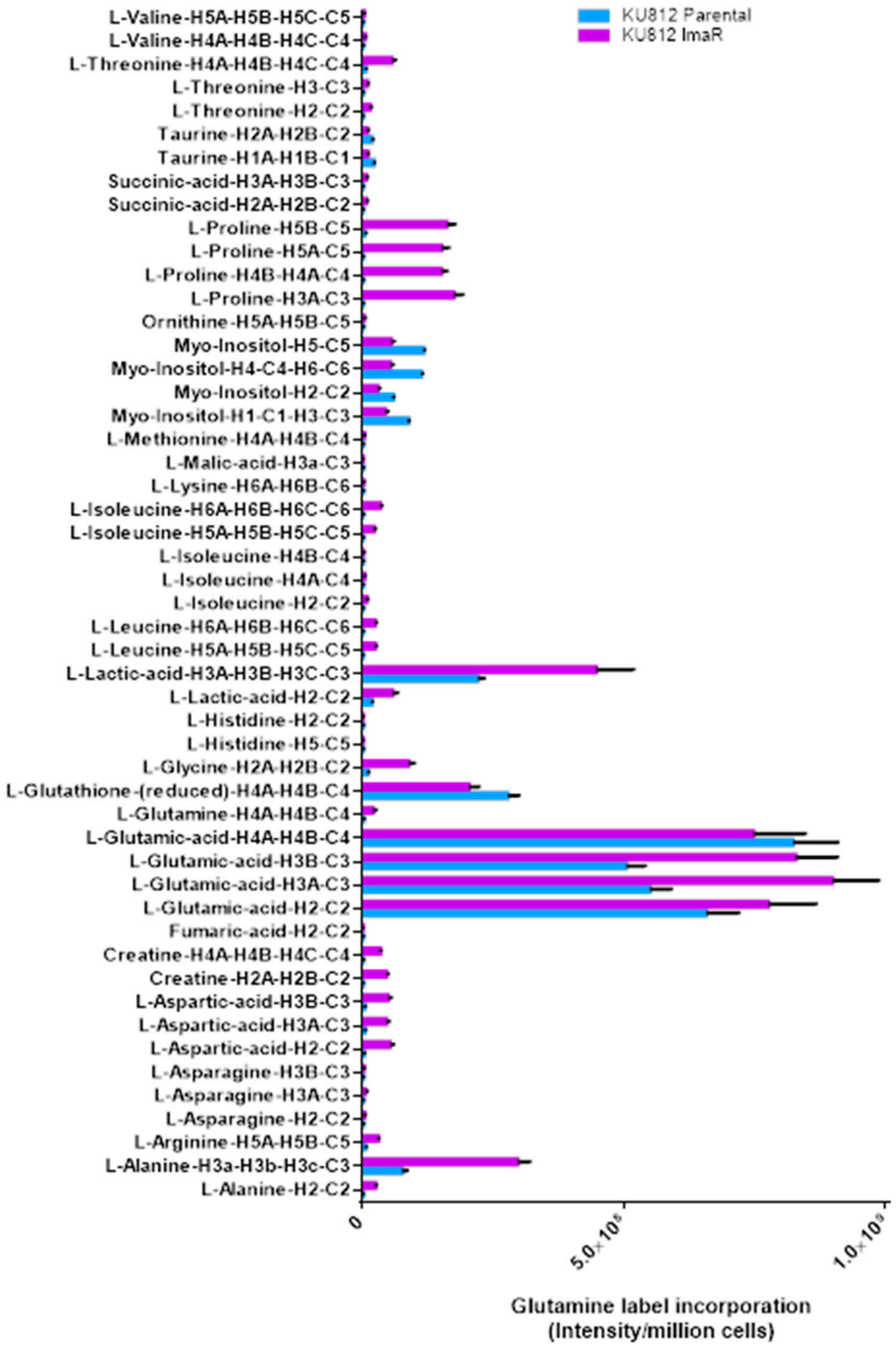

Figure S5. ^{13}C Glutamine label incorporation in KU812 Parental and ImaR cells under normoxia measured by NMR. Intensity values were normalized by million cells. Figure shows one representative experiment (mean±SD for n=3 replicates). Statistically significant differences between KU812 Parental and KU812 ImaR cells are not shown in this Figure; they are represented in Table S1**.** Abbreviations: ImaR, imatinib resistant; and N.D., non-detected metabolites.

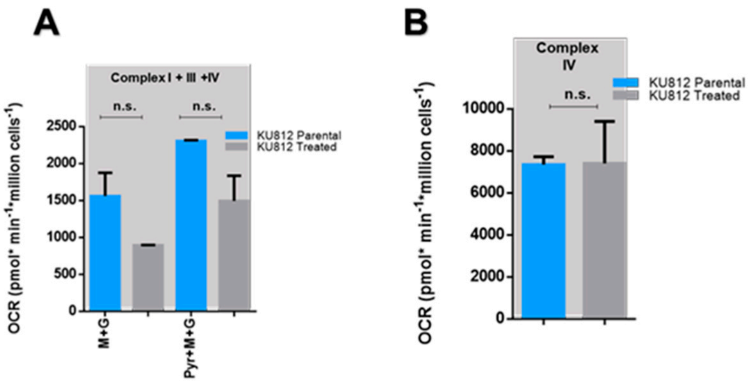

Figure S6. Activity of the different electron transport chain complexes of KU812 Imatinib-treated vs. KU812 Parental cells**.** OCR measurements of KU812 Parental and Treated (KU812 Parental cells treated with 80nM imatinib for 72 h) were determined by Oroboros Oxygraph-2k respirometer under normoxia. OCR were measured after sequential injections of substrates and inhibitors. For **A** (approach to study complex I activity): 2 mM malate + 5 mM ADP + 10 mM glutamate (M+G), and 5mM pyruvate (P). For **B** (complex IV activity): 5 mM Ascorbate + 5 mM ADP + 0.5 mM tetramethyl-p-phenylendiamine dihydrochloride (TMPD). Data were normalised by cell number. Data are provided as mean ± SD of n = 2 and significance was determined by two-tailed independent sample Welch's t-test. Statistically significant differences between KU812 P and Treated cells are indicated as $p \geq 0.05$ (n.s. = non-significant differences).

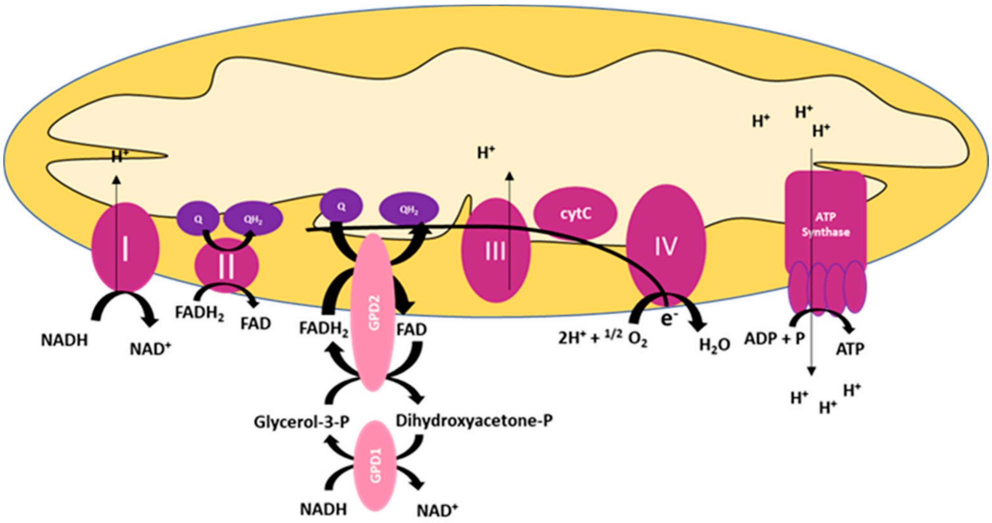

Figure S7. Diagram of the oxidative phosphorylation and glycerol-3-Phosphate shuttle.

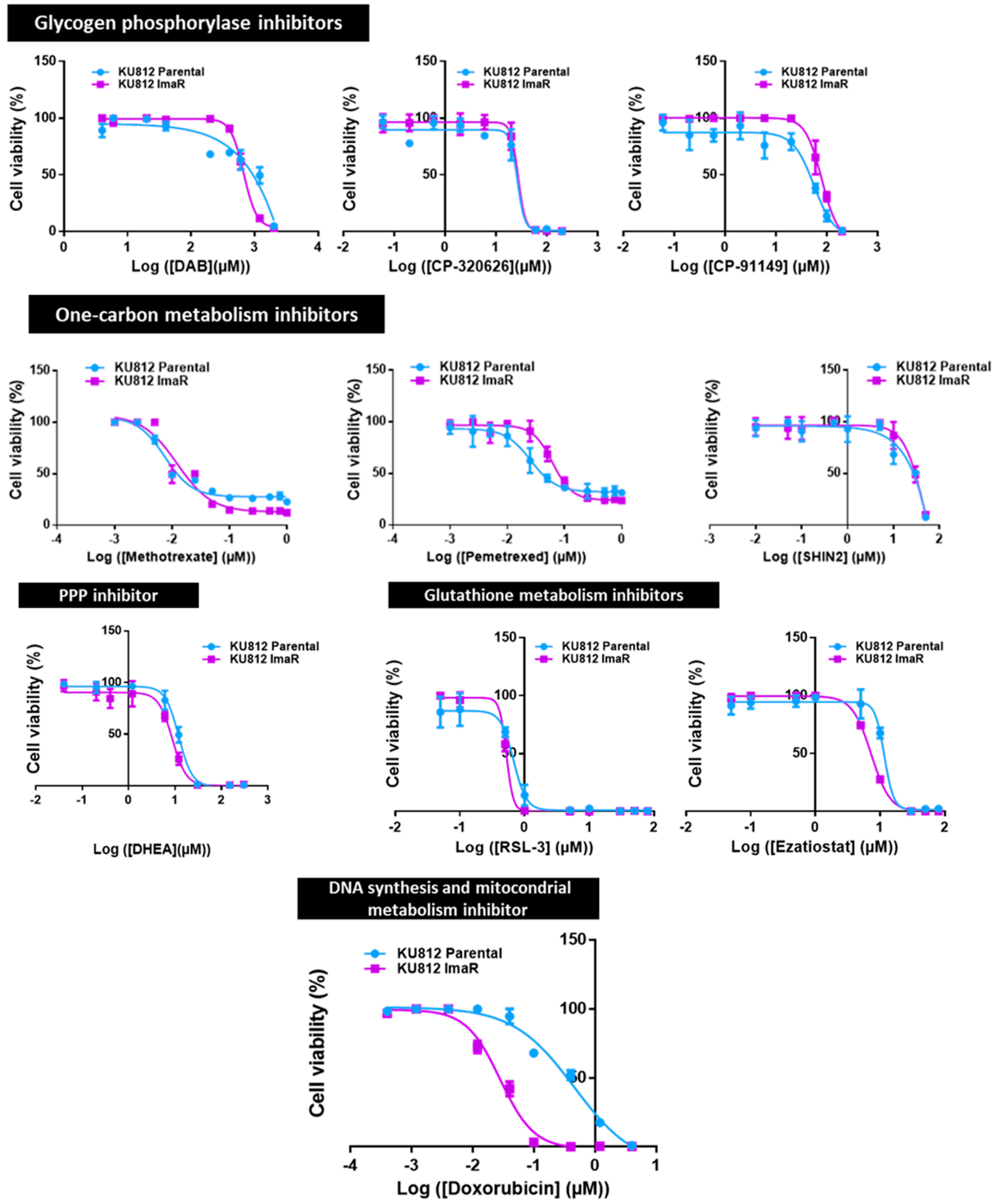

Figure S8. Effects of different metabolic inhibitors and AML conventional drugs in KU812 Parental and ImaR cell viability in normoxia. KU812 parental and ImaR cells were incubated 72 h with DMSO (vehicle control) or the log concentrations of the inhibitors shown. IC50s shown in Table 1. Figure shows one representative experiment (mean ± SD for n = 3) of n = 3 independent experiments.

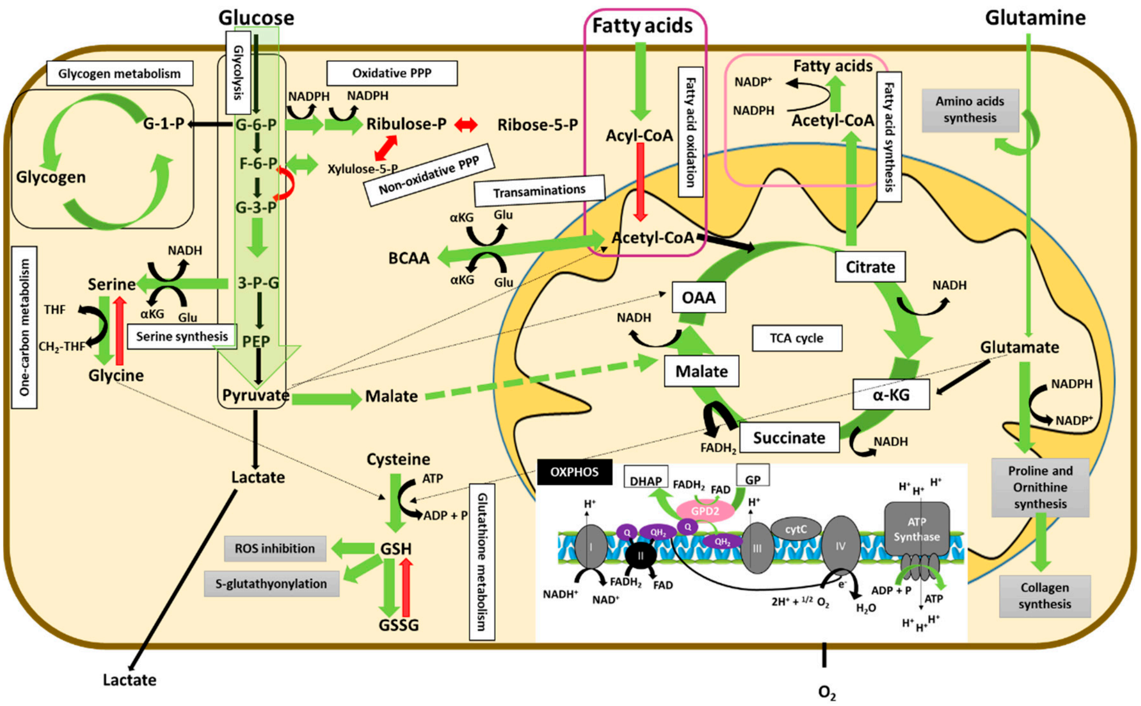

Figure S9. Schematic diagram of the metabolic reprogramming upon TKI resistance acquisition. Upregulated pathways are highlighted in green and downregulated pathways are highlighted in red.

Table S1. Exchange flux values of KU812 Parental and ImaR in normoxia after specific incubation times (Parental Norm = 96 h, ImaR Norm = 48 h, Parental Hyp = 72 h and ImaR Hyp = 24 h). Exchange flux values were obtained using Biocrates kit. Table shows the values of one representative experiment (mean ± SD for n = 3 replicates). Cysteine was not shown due to the inability of Biocrates kit to measure it.

EXTRACELLULAR FLUXES NORMOXIA					
Kpc (nmol*millioncell-1*h-1)					
	KU812 Parental		KU812 ImaR		
	Amino acids				
	Mean	SD	Mean	SD	pvalue
**Ala**	5.02	0.92	3.72	0.52	0.101
**Arg**	-0.65	0.25	-3.87	4.05	0.365
**Asn**	-0.26	0.02	-0.61	0.49	0.394
**Asp**	0.02	0.12	-0.41	0.13	0.013
**Cit**	0.01	0.00	-0.10	0.04	0.006
**Gln**	-9.12	0.24	-22.44	4.79	0.034
**Glu**	2.63	0.65	3.97	0.38	0.036
**Gly**	0.25	0.19	0.39	0.28	0.492
**His**	-0.21	0.10	-0.46	0.20	0.128
**Ile**	-1.43	0.09	-2.86	0.39	0.003
**Leu**	-1.93	0.13	-4.13	0.68	0.005
**Lys**	-1.25	0.12	-2.52	0.58	0.020
**Met**	-0.32	0.01	-0.92	0.17	0.004
**Orn**	0.61	0.09	1.80	0.60	0.028
**Phe**	-0.45	0.04	-1.11	0.23	0.008
**Pro**	0.20	0.15	2.47	0.27	0.000
**Ser**	-1.79	0.11	-5.05	0.89	0.003
**Thr**	-0.52	0.02	-1.91	0.68	0.025
**Trp**	-0.12	0.01	-0.25	0.09	0.062
**Tyr**	-0.38	0.06	-1.01	0.09	0.001
**Val**	-1.17	0.11	-2.56	0.47	0.007
Polyamines					
**Putrescine**	-4E-05	4E-05	-5E-04	2E-04	0.010
**Spermidine**	-7E-06	2E-05	2E-05	4E-05	0.362
**Spermine**	-7E-05	2E-05	-8E-04	1E-03	0.409

Table S2. Exchange flux values of KU812 Parental and ImaR in hypoxia (1% O_2) after specific incubation times (Parental Norm = 96 h, ImaR Norm = 48 h, Parental Hyp = 72 h and ImaR Hyp = 24 h). Exchange flux values were obtained using Biocrates kit. Table shows the values of one representative experiment (mean ± SD for *n* = 3 replicates). Cysteine was not shown due to the inability of Biocrates kit to measure it.

EXTRACELLULAR FLUXES HYPOXIA					
Kpc (nmol*millioncell-1*h-1)					
	KU812 Parental		KU812 ImaR		
	Amino acids				
	Mean	SD	Mean	SD	pvalue
Ala	3.19	0.51	1.85	0.63	0.045
Arg	-1.95	0.03	10.68	6.07	0.099
Asn	-0.86	0.88	0.32	0.57	0.308
Asp	-0.36	0.30	1.06	1.07	0.091
Cit	-0.02	0.00	0.09	0.18	0.356
Gln	-6.39	1.10	6.54	15.92	0.358
Glu	0.90	0.71	7.01	3.50	0.041
Gly	1.32	0.31	3.44	1.29	0.359
His	-0.02	0.09	0.41	0.68	0.336
Ile	-0.42	0.33	-1.00	1.10	0.433
Leu	-0.83	0.22	-1.97	1.02	0.130
Lys	-0.24	0.64	-1.49	0.87	0.115
Met	0.08	0.01	-0.01	0.73	0.871
Orn	2.15	0.51	1.90	0.66	0.639
Phe	0.09	0.25	-0.54	0.30	0.049
Pro	0.46	0.22	1.47	0.44	0.023
Ser	-1.58	0.26	-8.33	0.95	0.000
Thr	-0.25	0.37	0.13	1.51	0.695
Trp	0.02	0.02	-0.25	0.18	0.054
Tyr	0.13	0.29	-0.93	0.31	0.012
Val	0.03	0.53	-1.51	0.81	0.051
Polyamines					
Putrescine	4E-04	2E-04	-2E-03	3E-04	0.001
Spermidine	-1E-06	2E-05	5E-05	2E-04	0.739
Spermine	-3E-05	3E-05	1E-04	2E-04	0.315

Table S3. Intracellular content of KU812 Parental and ImaR in normoxia after 24 h incubation time. Intracellular contents were obtained using Biocrates kit. Table shows the values of one representative experiment (mean ± SD for *n* = 3 replicates). Cysteine was not shown due to the inability of Biocrates kit to measure it. Abbreviations: D, discarded.

INTRACELLULAR CONTENT NORMOXIA					
nmol/mg protein					
	KU812 Parental		KU812 ImaR		
	Amino acids				
	Mean	SD	Mean	SD	pvalue
**Ala**	271.86	130.35	448.71	198.69	0.267
**Arg**	89.71	14.48	96.74	48.08	0.820
**Asn**	253.43	14.36	500.37	178.44	0.075
**Asp**	180.60	72.28	222.23	39.51	0.523
**Cit**	D	D	D	D	D
**Gln**	1162.32	500.57	2141.94	847.04	0.160
**Glu**	D	D	D	D	D
**Gly**	275.13	142.35	768.70	235.22	0.057
**His**	26.28	3.18	47.00	28.66	0.281
**Ile**	67.55	13.16	138.49	32.39	0.025
**Leu**	55.69	8.21	170.55	50.71	0.018
**Lys**	23.24	4.06	20.24	1.44	0.295
**Met**	19.15	3.70	52.75	28.28	0.111
**Orn**	6.84	0.59	38.37	11.96	0.010
**Phe**	18.59	1.46	34.06	6.78	0.018
**Pro**	43.54	1.65	D	D	D
**Ser**	39.94	14.85	81.03	69.67	0.374
**Thr**	65.16	10.89	134.42	19.08	0.005
**Trp**	3.42	1.48	8.04	1.58	0.021
**Tyr**	30.27	3.48	60.51	21.60	0.075
**Val**	22.63	5.55	21.91	10.02	0.918
Polyamines					
**Putrescine**	23.63	7.46	4.22	0.90	0.011
**Spermidine**	31.66	2.06	26.32	7.98	0.324
**Spermine**	15.49	0.66	9.63	2.82	0.025

Table S4. Intracellular content of KU812 Parental and ImaR in hypoxia (1% O_2) after 24 h incubation time. Intracellular contents were obtained using Biocrates kit. Table shows the values of one representative experiment (mean ± SD for *n* = 3 replicates). Cysteine was not shown due to the inability of Biocrates kit to measure it. Abbreviations: D, discarded.

INTRACELLULAR CONTENT HYPOXIA					
nmol/mg protein					
	KU812 Parental		KU812 ImaR		
	Amino acids				
	Mean	SD	Mean	SD	pvalue
Ala	196.81	70.77	555.71	205.39	0.267
Arg	124.89	82.72	168.62	8.77	0.820
Asn	374.27	155.35	584.22	159.18	0.075
Asp	41.06	15.07	176.95	111.59	0.523
Cit	D	D	D	D	D
Gln	2102.55	122.34	2939.16	475.32	0.160
Glu	D	D	D	D	D
Gly	341.37	25.92	1847.53	898.01	0.057
His	50.56	17.24	85.98	22.96	0.281
Ile	124.73	37.62	306.43	65.23	0.025
Leu	138.61	18.59	352.46	29.64	0.018
Lys	68.98	24.70	89.94	44.29	0.295
Met	36.54	14.07	84.08	35.11	0.111
Orn	60.62	32.88	52.32	16.46	0.010
Phe	33.73	3.72	75.25	11.72	0.018
Pro	223.72	47.62	D	D	D
Ser	99.18	67.01	107.43	8.54	0.374
Thr	98.19	10.44	247.54	98.36	0.005
Trp	8.06	1.54	14.28	5.59	0.021
Tyr	55.90	13.18	113.93	32.12	0.075
Val	66.16	32.79	104.60	37.65	0.918
Polyamines					
Putrescine	22.53	9.31	1.05	0.19	0.011
Spermidine	41.91	0.44	59.18	36.97	0.324
Spermine	19.49	6.13	25.64	13.60	0.025

Original Western Blot:

Figure 3.E

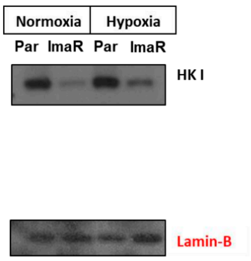

Figure 4.B

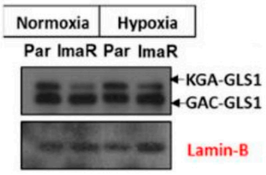

Membrane 1:

HKI and the two isoforms of GLS were performed in the same membrane.

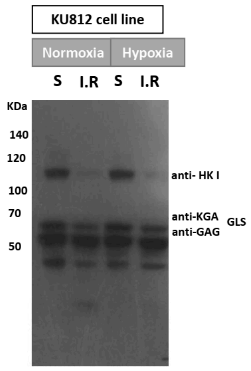

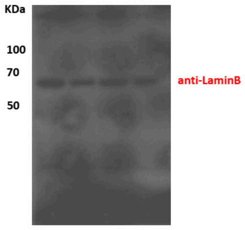

ORIGINAL SCANS

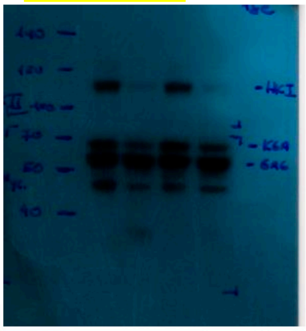

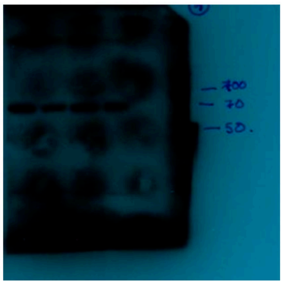

Figure 3.E

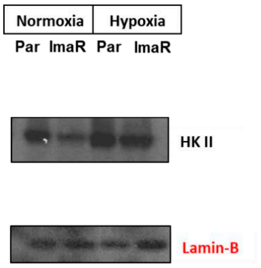

Membrane 2:

HKII membrane.

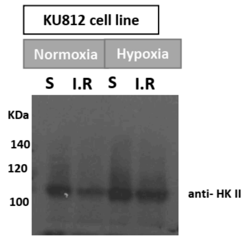

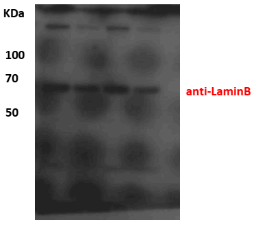

ORIGINAL SCANS

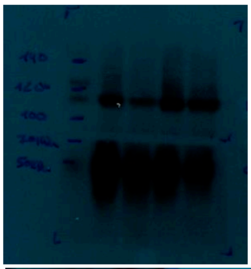

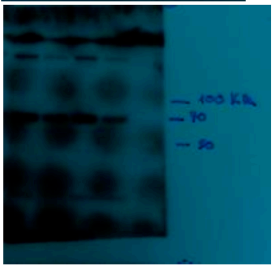

Figure 3.E

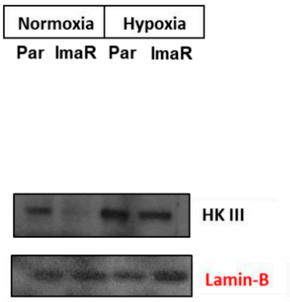

Membrane 3:

HKIII membrane.

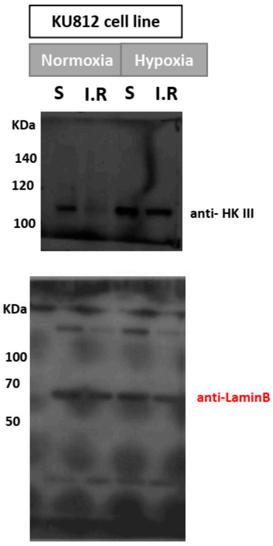

ORIGINAL SCANS

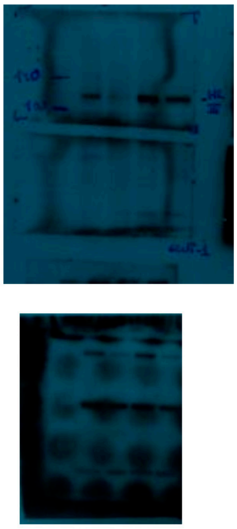

Membrane 4:

TKT membrane.

Figure 3.E

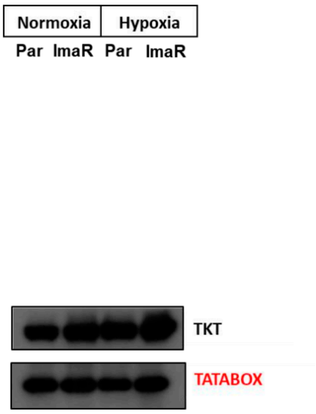

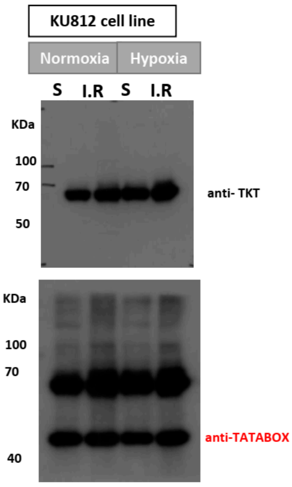

ORIGINAL SCANS

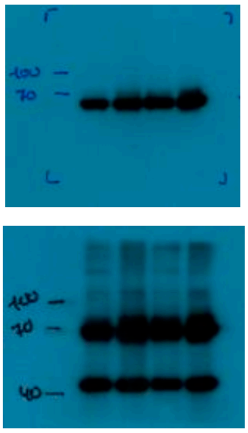

Membrane 5:

P5CS and GL-Syn proteins were performed in the same membrane.

Figure 4.B

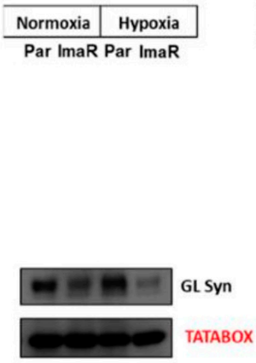

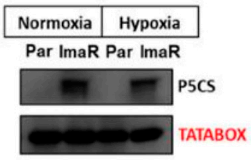

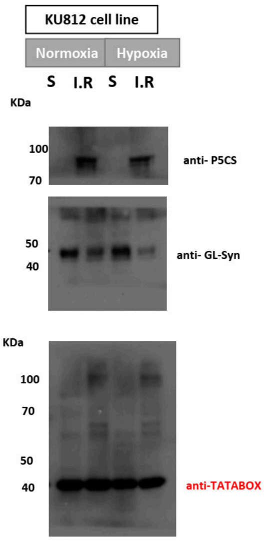

ORIGINAL SCANS

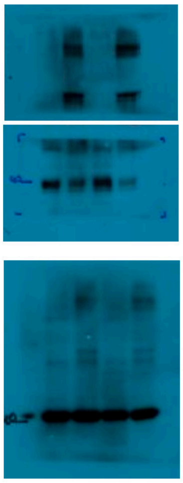

Membrane 6:

GLS2 protein

Figure 4.B

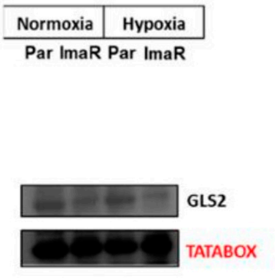

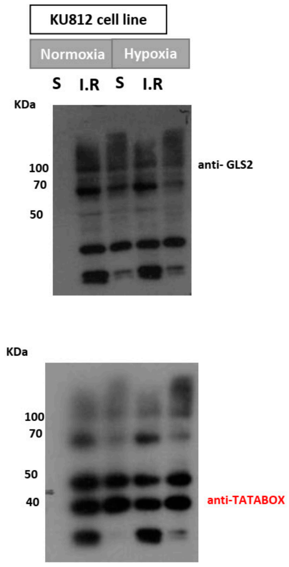

ORIGINAL SCANS

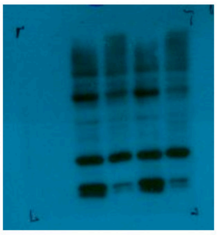

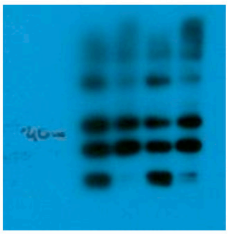

Membrane 7:

PRODH protein

Figure 4.B

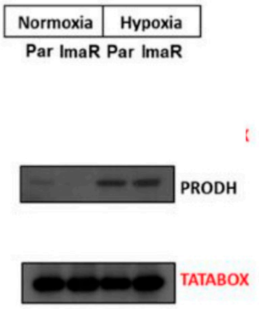

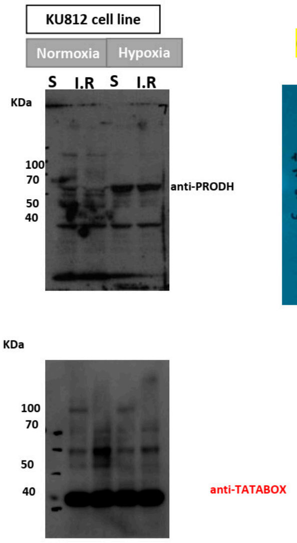

ORIGINAL SCANS

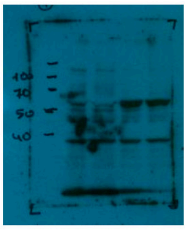

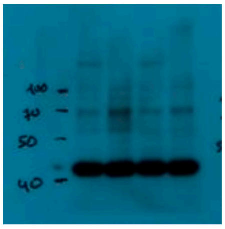

Membrane 8:

PYCR protein

Figure 4.B

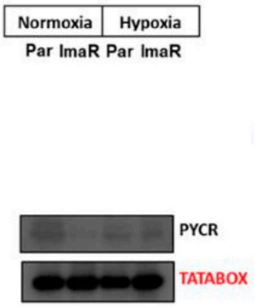

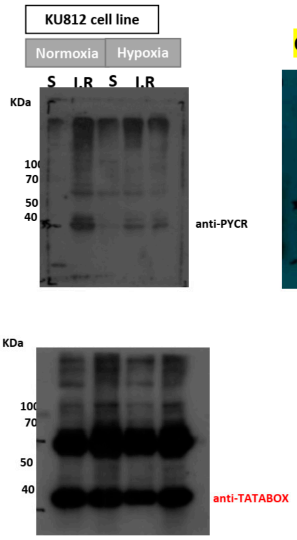

ORIGINAL SCANS

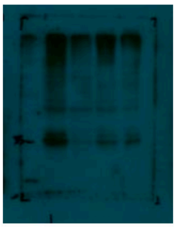

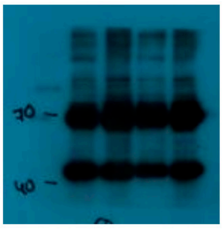

Supplement: Supplementary file 1 [file cancers-12-03443-s001.zip › cancers-980787 supp proof.pdf]
